# Mathematical modelling of diurnal regulation of carbohydrate allocation by osmo-related processes in plants

**DOI:** 10.1098/rsif.2014.1357

**Published:** 2015-03-06

**Authors:** Alexandra Pokhilko, Oliver Ebenhöh

**Affiliations:** 1 Institute for Complex Systems and Mathematical Biology, University of Aberdeen, Meston Building, King's College, Aberdeen, UK; 2 Cluster of Excellence on Plant Sciences (CEPLAS), Heinrich-Heine-University, Universitätsstraße 1, Dusseldorf 40225, Germany

**Keywords:** mathematical modelling, carbohydrate metabolism, plant physiology, circadian clock, systems biology

## Abstract

Plants synthesize sucrose in source tissues (mainly mature leafs) and supply it for growth of sink tissues (young leafs). Sucrose is derived from photosynthesis during daytime and from starch at night. Because the diurnal regulation of sucrose fluxes is not completely understood, we built a mathematical model designed to reproduce all key experimental observations. For this, assumptions were made about the molecular mechanisms underlying the regulations, which are all motivated by experimental facts. The key regulators in our model are two kinases (SnRK1 and osmo-sensitive kinase OsmK) under the control of the circadian clock. SnRK1 is activated in the night to prepare for regularly occurring carbon-limiting conditions, whereas OsmK is activated during the day to prepare for water deficit, which often occurs in the afternoon. Decrease of SnRK1 and increase of OsmK result in partitioning of carbon towards sucrose to supply growing sink tissues. Concomitantly, increasing levels of the growth regulator trehalose-6-phosphate stimulates the development of new sink tissues and thus sink demand, which further activates sucrose supply in a positive feedback loop. We propose that OsmK acts as a timer to measure the length of the photoperiod and suggest experiments how this hypothesis can be validated.

## 1. Introduction

### 1.1. Background

Sucrose is the main transported sugar in plants. It is largely synthesized in mature leaves, so-called source tissues, from where it is transported to sink tissues to support growth. During the day, photosynthesis-derived carbon is partly used for sucrose production and partly for starch accumulation. These transiently stored starch reserves are then used to facilitate a continued sucrose production during the night [1,2]. Apparently, carbon fluxes determining which fraction of the assimilated carbon is used for starch accumulation and how fast the stored starch is then degraded during the night, must be regulated in order to ensure a continuous supply of sucrose and to avoid starvation near the end of the night. It is indeed observed that synthesis and degradation of starch are dynamically adjusted to changing photoperiods, such that starch is synthesized faster in short than in long days [1,3–5], demonstrating that plants somehow know there is less time to prepare for the longer night. Starch degradation rates are regulated in the opposite way, with degradation being slower in short days. Interestingly, observed degradation rates in wild-type *Arabidoposis thaliana* plants are optimized to ensure that premature depletion of starch is avoided but only little starch is retained at the end of the night [4,6]. Logically, due to the lower amount of total available carbon, in short days less sucrose can be consumed by sink tissues and therefore plant growth is slower [5]. Interestingly however, sucrose supply to sink tissues is proportional to total assimilated carbon both during day and night [5]. A remarkable finding by the pioneering ‘early dusk’ experiments [6] was that plants are capable of immediately adjusting starch degradation rates to an unexpected early darkness, as long as the total circadian cycle remains at 24 h.

These observations make it evident that plants can somehow measure the durations of light and dark periods and use this information to regulate carbon fluxes. Further, the strong dependence of a correct starch turnover on a T-cycle (total period of one day/night cycle) of around 24 h clearly demonstrates the involvement of the circadian clock in this process [6,7]. However, how exactly day and night lengths are measured and which molecular mechanisms are responsible for processing this information with cues from the clock, is still largely unknown.

Complicated interaction networks, such as that governing the diurnal regulation of carbon allocation, are too complex to be understood by simple intuition and qualitative reasoning. Therefore, in modern molecular biology, mathematical models become increasingly important because they provide a way to systematically analyse the behaviour of a complex system and to explain observations, which result as emergent system properties [8,9]. Recently, we have presented a mathematical model in which we have started to derive hypotheses about the molecular basis of the diurnal regulation of carbon fluxes in the model plant *A. thaliana* ([10], here called P2014). The P2014 model contained three modules, representing carbon metabolism, the circadian clock and a regulatory module integrating environmental and circadian cues to adjust metabolic fluxes. A central component of P2014 was the key regulatory role of the *SnRK1* kinase (SNF1-related kinase 1). Briefly, we proposed, based on experimental data [11,12], that *SnRK1* inhibits sucrose synthesis enzymes under conditions in which carbon is sparse (termed ‘carbon deficit’ conditions, such as in short days). We also proposed that the molecular mechanism to sense carbon deficit and other circadian cues by *SnRK1* kinase is provided by its β subunit, AKINβ1 (in the model described by the variable *β*), which accumulates in darkness. With these assumptions, the P2014 model could provide data-driven, testable hypotheses about the biochemical mechanisms to regulate starch synthesis. However, molecular details of other important mechanisms remained purely hypothetical. For example, it was necessary to include a daytime signal (a ‘timer’ *α*), which accumulates during the day and sets the rate of starch degradation at night. However, the molecular identity of the timer *α* remained unclear. Further, in the P2014 model, it was necessary to speculate on a component (termed *D*), which facilitates the regulation of sucrose supply in source tissues by demand from sinks [13,14].

### 1.2. Novel hypotheses and key model improvements

Re-interpretation of existing data and novel experimental findings made it possible to derive hypotheses on the molecular nature of these hitherto purely speculative components. Thus, the main goal of the current work is to provide an updated and considerably enhanced mathematical model, which (i) reflects the old and novel findings and (ii) provides an explanation of experimental data, in particular diurnal turnover of starch, sucrose and trehalose 6-phosphate (*T6P*) and (iii) predicts carbon fluxes under various perturbations to test the newly proposed molecular regulation mechanisms. In the following, we outline the data-driven hypotheses, which enter our updated model.

The following observations led to our proposition that osmo-sensitive kinases (*OsmK*) play a key role in photoperiod measurements in plants. It may seem surprising why response factors to water limitation mediated by *OsmK* are related to the timing of metabolic processes, but in fact there exists a high regularity of the occurrence of water deficit: during the day, *A. thaliana* (and other C3 plants) open their stomata pores to access carbon dioxide from the air. However, this also promotes water evapouration. While the level of water deficit naturally varies from day to day, it is most likely to occur during the hot phases of the early afternoon. Plants prepare for this regularly recurring limitation by activating *OsmK*s in the afternoon through the circadian clock [15,16]. These *OsmK*s accelerate accumulation of sugars via activation of key enzymes of sucrose synthesis, such as sucrose-phosphate synthase (SPS) [17,18], which is believed to help plants retain water [19]. This notion is supported by the observation that sucrose production is also increased in plants exposed to water deficit [20–22]. The observed daily rhythms of SPS activity under normal conditions [23] are most likely related to the diurnal activation of *OsmK* kinases, explaining the accumulation of sugars in plants during the day [5], in parallel to the expected water loss [24]. The activated *OsmK*s include Ca^2+^-dependent and Ca^2+^-independent kinases, such as SnRK2 and SnRK3 [25,26]. Interestingly, the circadian regulation includes Ca^2+^-dependent responses, since release of Ca^2+^ from intracellular stores, mediated by circadian accumulation of the signalling molecule ADP-ribose, has a pronounced circadian peak in the afternoon [27,28]. These factors cause a continuous increase of *OsmK* activities during the day and thus provide a means to measure the photoperiod.

The parallel changes of day and night sucrose supply under different photoperiods [5] suggest that the regulation of sucrose synthesis during the day and starch degradation at night possess a common underlying mechanism. Indeed, like sucrose synthesis, starch degradation is activated by processes related to water limitation [20,29,30]. Signalling the water status to starch degradation includes a quick adjustment of the chloroplastic to the cytosolic osmotic state [31]. Similarly, the circadian changes in Ca^2+^ levels are transmitted from cytosol to chloroplast by channelling Ca^2+^ into chloroplasts after dusk [27]. The changes in osmotic state and Ca^2+^-dependent processes modulate starch degradation in various ways, including phosphorylation of key starch-degrading enzymes in the chloroplast, such as GWD, which catalyses the initial phosphorylation of the starch granule surface [32–35] and transcriptional upregulation of starch-degrading enzymes by Ca^2+^-dependent and *OsmK* kinases [36,37]. Therefore, it is reasonable to assume that *OsmK* and Ca^2+^-dependent kinases directly or indirectly activate starch degradation. The afternoon increase of the activities of these kinases makes them perfect candidates for the diurnal activator of starch degradation *α* in the P2014 model.

Our second hypothesis states that *T6P* acts as the demand regulator *D*, which signals the sink's demand to the source tissues. In general, demand for sucrose depends on the growth rate, which is activated by T6P via stimulation of cell differentiation in fast growing meristematic tissues, where T6P levels are up-regulated [38–40]. Moreover, mutant studies show that absence of T6P results in a dramatic reduction of meristematic regions of growing tissues, resulting in growth retardation and drastically reduced plant sizes [38]. The stimulatory effect of T6P on sucrose consumption by sink tissues makes T6P a good candidate for the demand regulator *D*. Existence of a feedback signal from sink demand to source tissues has been demonstrated in various experiments, in which the sucrose demand by sinks was altered, e.g. by defoliation (removal of the source leaves) or a sudden change in temperature [13,14]. Increasing demand relative to supply led to a simultaneous upregulation of the two key enzymes of sucrose synthesis, SPS and cFBPase (cytosolic fructose-1,6-bisphosphatase) and thus of sucrose supply by source tissues [13,14]. The biochemical details for the quick (approx. 10 min [14]) propagation of the signal from sink to source tissues require further research, but a possible scenario includes propagation by waves of changing sugar and ion concentrations, initiated by increased demand and mediated by various phloem transporters connecting source and sink tissues. These events trigger the activation of *OsmK* and sucrose synthesis, followed by loading sucrose into the phloem at source leaves [41–44]. Interestingly, it was found that under many experimental conditions, the concentrations of *T6P* and sucrose are highly linearly correlated [39,45,46]. The exact mechanisms underlying this observation are again not completely understood, however there is evidence for the involvement of *SnRK1*. SnRK1 is inhibited by sugar phosphates in sink tissues [47–49], and itself can inhibit components of the *T6P* synthase (TPS) complex [50–53]. This duplicate role of T6P as a sensor of sugars and an activator of sugar consumption further underpin the hypothesis that T6P is a good candidate for the demand regulator *D*.

**Figure 1.**
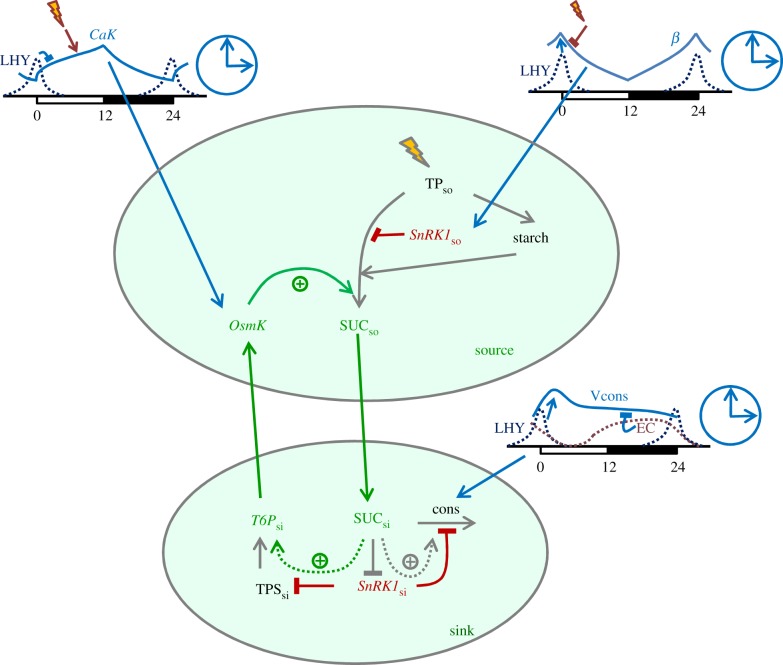
Schematic illustration of the principal diurnal and circadian mechanisms regulating carbon fluxes in the model. Triose-phosphates (TP_so_), fixed during the day by photosynthesis (flash) are partitioned to synthesize sucrose and starch. Sucrose synthesis is inhibited by *SnRK1* kinase and activated by osmo-sensitive kinase *OsmK. SnRK1* senses rhythmic changes in carbon deficit via AKINβ1 (*β*), whereas *OsmK* senses rhythmic changes in water deficit via Ca^2+^-dependent kinase (*CaK*). Both diurnal sensors *β* and *CaK* are regulated by the clock (*β* is activated by the clock protein LHY and *CaK* is inhibited by LHY) and light (*β* is inhibited by light and *CaK* is activated by light, shown by flashes). Thus, sucrose synthesis during the day is increasing due to increased *CaK* and decreased *β*. Sucrose is then exported and consumed by sink tissues, where it accelerates the synthesis of *T6P* (*T6P*_si_) by a double negative regulation: sucrose inhibits *SnRK1*, which in turn inhibits TPS. *T6P*_si_ accelerates the development of sink tissues, thus increasing sink demand for carbon during the day. Increased demand in turn activates *OsmK*, creating a positive feedback loop activating source supply by sink demand. At night, *OsmK* accelerates starch degradation and thus upregulates sucrose production. Therefore, activation of sucrose supply during the day increases sink demand, which in turn increases *OsmK* and upregulates starch degradation and thus sucrose supply at night. The mutual positive interactions between source supply and sink demand, which are shown by green lines, are gated by the clock via *CaK*. Positive effects of *OsmK* on sucrose fluxes are opposed by *SnRK1* (red). *SnRK1* further inhibits sugar consumption in sink tissues. This results in an activation of sugar consumption by high sugar levels by double negative regulation: sugars inhibit *SnRK1*, which in turn inhibits sugar consumption. Consumption of sugars by sink tissues is additionally regulated by the clock via activation by LHY and inhibition by the EC.

Apart from modifying the P2014 model to include the two key hypotheses detailed above (figure 1), it was further improved to reproduce a broader spectrum of carbon stress conditions. Under normal conditions, it is assumed that the starch degradation rate is set at dusk according to the starch level and the time to dawn, in order to provide a nearly constant degradation rate [1,10,54]. This temporal gating is probably related to the formation of multi-protein starch-degrading complexes soon after dusk [1]. However, starch degradation was observed to slow down in the second part of the night under carbon stress, when sugars reach critically low levels [6,55]. A possible molecular mechanism is the downregulation of β-amylase abundance by sugar starvation [55,56]. The resulting decrease of carbon supply to sinks reduces sucrose consumption and allows the little carbon reserves to last longer. Therefore, it seems that the maximal rate of starch degradation is indeed set at dusk, but it might be further decreased at night in case of starvation. Low sugar levels also upregulate transcription of various subunits of *SnRK1*, for example AKINβγ, which further activates *SnRK1* [57,58]. These additional mechanisms sensing low sugar levels and adapting carbon fluxes and starch degradation to severe carbon limitations have now been introduced in the model.

As discussed above, the circadian clock is important to allow anticipation of regularly re-occurring rhythmic changes in the environment, such as a shortage of carbon near the end of the night or water limitation in the afternoon. It has further been shown that the clock regulates the consumption of sugars by growing tissues by gating the transcription of key growth activators (PIF4 and PIF5, [59]). This clock-regulated sugar consumption is now included in the model.

With the improvements described above, our model is capable of describing existing experimental dynamics of starch, sucrose and *T6P* levels under various conditions, thus the range of experimental observations, which can now be explained, is considerably enhanced. Moreover, we employ the model to make novel predictions on the plant's response to new perturbations, which allow the design of further experiments to test the proposed regulatory mechanisms and thus to increase our basic understanding of the regulatory principles of carbon allocation in plants.

## 2. Model description

The metabolic module of the model was inherited from P2014, while the diurnal regulation module was substantially revised. The model consists of 30 ordinary differential equations (21 equations in the metabolic module and nine equations in the diurnal module). The detailed description of the model is provided in electronic supplementary material, S1. Briefly, the model describes diurnal changes in carbon metabolism in source and sink tissues. In source tissues, carbon is fixed in chloroplasts during the day to yield triose-phosphates (TP), which are partitioned between starch synthesis in chloroplasts and sucrose synthesis in the cytosol (electronic supplementary material, figure S1). Sucrose is further exported and consumed by sink tissues. At night, starch degradation provides carbon for sucrose synthesis, which again is used for consumption by sink tissues (electronic supplementary material, figure S1). The fluxes of carbon are adjusted to diurnal conditions by a small subsystem of diurnal regulators, which integrate light and clock signals to set activities of the key enzymes according to the environmental conditions. Based on experimental evidence, the P2014 scheme of diurnal regulations was substantially revised (figure 1) and essential mechanisms and connections were included, allowing the specification of actual molecular candidates for previously hypothetical compounds (electronic supplementary material, figure S2). In our model, the key enzymes of the sucrose synthesis pathway, cFBPase and SPS, are inhibited by *SnRK1* kinase, similar to P2014. *SnRK1* is activated by its β subunit AKINβ1 (β), which is upregulated by darkness and the circadian protein LHY (figure 1, [10]). Thus, β measures the total duration of the night and activates *SnRK1* at night, which leads to an inhibition of sucrose synthesis and acceleration of starch synthesis in short days, in agreement with experimental data [3,10]. Therefore, similar to the P2014 model, *SnRK1* provides adaptation of the starch synthesis rate to the conditions encountered in the previous day. In the current model, we further included the inhibition of *SnRK1* by sugars in sink tissues, which is relevant under normal, non-stress, conditions [47–49] and transcriptional upregulation under carbon stress [57,58], providing an additional, but slower, increase of *SnRK1* under stress.

Motivated by the experimental observations detailed in the Introduction, we included in the current model the regulation by osmo-sensitive kinase *OsmK*. In the model presented here, sucrose synthesis enzymes are also activated by *OsmK*, which gradually increases during the day ([15,16]; electronic supplementary material, figure S3a). *OsmK* integrates two types of diurnal signals in our model (figure 1). The first signal is associated with anticipated water deficit during the day, presumably mediated by the circadian release of Ca^2+^ and the activation of Ca^2+^-dependent kinase (*CaK*). *CaK* is activated by light and by the clock in the afternoon (figure 1; electronic supplementary material, figure S3a). The second signal originates from the sink's demand for carbon. This demand is proportional to *T6P* concentrations in sinks (*T6P*_si_), an assumption based on the fact that *T6P* activates cell differentiation in sink tissues [38,39], which increases the amount of carbon required for cell growth and expansion (so-called sink strength). Diurnal changes of *T6P* levels were described in our model through changes in TPS activity, which presumably is inhibited by *SnRK1* [50,51] and activated by light [45,60]. The double negative connection through inhibition of sink *SnRK1* by sugars and TPS by *SnRK1* results in activation of *T6P*_si_ synthesis by sugars as described in Results (figure 1). In summary, *SnRK1* (activated by carbon deficit) and *OsmK* (activated by water deficit) have the opposite effect on carbon fluxes in our model: *SnRK1* inhibits sucrose supply, while *OsmK* activates sucrose supply to sink tissues during the day (figure 1).

At night, starch is the main source of carbon and consequently its degradation is subject to a strong diurnal regulation. As described in the Introduction, we assumed that osmotic kinase *OsmK* activates sucrose flux not only during daytime, but also at night, through the acceleration of starch degradation in chloroplasts. It was assumed that under normal, non-stress conditions starch degradation rate is set at dusk to be proportional to the starch level and to the activity of *OsmK* kinase, which plays the role of the timer *α* in P2014. Setting the starch degradation rate at dusk was described similarly to P2014 with the model variable *X*, which increases during the day according to starch and *OsmK* levels and remains constant in the night (electronic supplementary material, figure S3d). Starch degradation may decrease under carbon stress, which was described through downregulation of β-amylase by a severe depletion of sugars [55,56]. Intuitively, it seems a reasonable strategy to fix a maximal rate of starch degradation at dusk, so that an unexpected increase of sink demand, for example through herbivore attack, would not result in a premature exhaustion of carbon before the end of the night [61].

**Figure 2.**
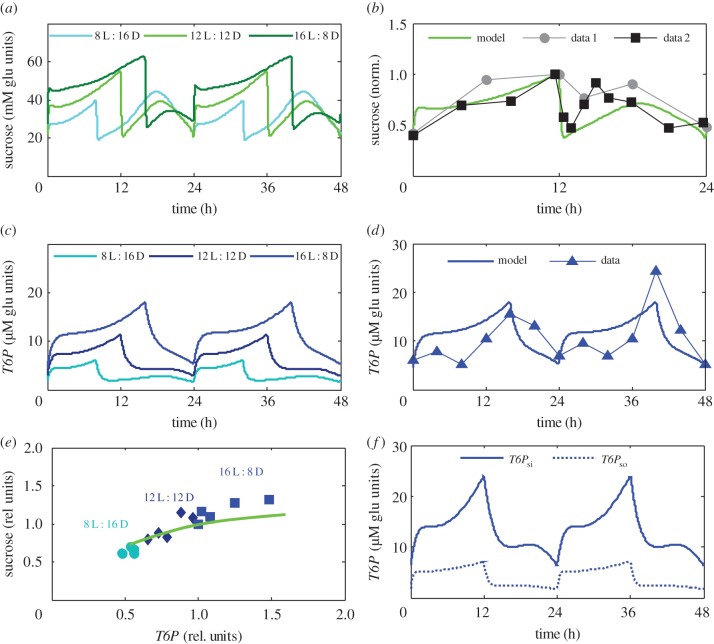
Diurnal profiles of sucrose and *T6P*. (*a*,*c*) Simulated diurnal kinetics of sucrose (*a*) and *T6P* (*c*) in *Arabidopsis* plants grown in 8 L : 16 D, 12 L : 12 D and 16 L : 8 D days (different colours). Total *T6P* and sucrose in a whole plant were calculated using both source and sink pools. (*b*) Experimentally observed and theoretically predicted sucrose kinetics for plants grown under 12 L : 12 D. Data points are redrawn from [66] (circles) and [46] (squares). To minimize the variation between different experiments and compare with the model, the data were normalized to the maximal sucrose level. (*d*) Experimentally observed and theoretically predicted *T6P* kinetics in plants grown under 16 L : 8 D. Data points are redrawn from [39]. (*e*) The inter-dependence of sucrose and *T6P* contents at the end of the day in the model (line) and in the data (symbols). Different sucrose and *T6P* levels were obtained by varying the duration of the light period. Data points are redrawn from [45] and correspond to different photoperiods: 8 L : 16 D, circles; 12 L : 12 D, diamonds; 16 L : 8 D, squares. (*f*) Source and sink pools of *T6P* in model simulations of plants grown under 12 L : 12 D diurnal cycle.

As in P2014, the consumption of sucrose in sink tissues is inhibited by *SnRK1* [10,62]. By contrast, in the new model version, *SnRK1* is also inhibited by sink sugars, resulting in a double negative regulation (figure 1). This motif represents an example of a feed-forward regulation, which is found in many biological systems [63,64]: increasing sugar levels will activate their consumption while decreasing levels will inhibit consumption (see Results). Consumption of sugars is further regulated by the clock through the key transcriptional activators of plant growth PIF4 and PIF5 [59]. We here explored how this affects the diurnal kinetics of sucrose accumulation. Transcriptional profiles of PIF4 and PIF5 in clock mutants suggest that these transcription factors are activated by the morning clock proteins LHY and CCA1 and inhibited by the so-called evening complex (EC), which acts at night and consists of the evening clock proteins ELF3, ELF4 and LUX [59]. Thus, we included in the model activation of sugar consumption by the LHY/CCA1 complex and inhibition by the EC (figure 1). In the model (figure 1), the LHY/CCA1 complex also contributes to the regulation of carbon partitioning (through *β*) and starch degradation (through *CaK*), which reflects the observed complex effects of *lhy*/*cca1* mutations on carbon metabolism [6,10]. By contrast, the EC only contributes to regulating consumption, which agrees with the observed mild metabolic phenotype of the strong circadian mutant *elf3* [65] (see Results). The differences between the current model and P2014 are schematically presented in electronic supplementary material, figure S2, which illustrates that certain oversimplifications and uncertainties were removed to provide better agreement with data. The model is still able to quantitatively describe all phenomena that were already correctly described in P2014 (electronic supplementary material, figures S4 and S5, S1). Moreover, the inclusion into the model of a significantly broader range of experimental facts led to an improvement in reproducing sucrose kinetics (figure 2*b*, and electronic supplementary material, figure S6a, S1). Importantly, we can now simulate correctly the diurnal kinetics of the essential plant development regulator *T6P* (see Results). Additionally, the description of the clock mutant *elf3* was substantially improved compared with P2014 (Results; figure 6, and electronic supplementary material, figure S6b,c, S1). Despite the wider range of reproduced data, the number of parameters in the diurnal regulation module did not greatly increase in the current model. The new equations describing *SnRK1*, *T6P*, *OsmK*, *CaK* and their effects on carbon fluxes and the equation describing consumption rate have only four more parameters compared to the respective equations for *SnRK1*, *D*, *α* and the consumption rate in P2014 (see electronic supplementary material, S1, for more details). The values of the model parameters are presented in electronic supplementary material, table S1. Parameters of the metabolic reactions were taken from existing literature (electronic supplementary material, table S1), while unknown parameters related to diurnal regulation were chosen to fit the diurnal data on starch, *T6P* and sucrose in wild-type *Arabidopsis* plants under various photoperiods [6,39,46,55,66]. In mutant simulations, the rates of production of mutated components were set to 0, as indicated in the respective figure legends. The system of ordinary differential equations was solved using MATLAB, integrated with the stiff solver ode15 s (The MathWorks, Cambridge, UK). A MATLAB version of the model is supplied in electronic supplementary material, S2.

## 3. Results and discussion

### 3.1. Diurnal kinetics of sucrose and T6P in plants

In agreement with experimental observations [46,66], the model simulations show sucrose accumulation during the day (figure 2*a*,*b*). The afternoon increase in sucrose levels is explained by activation of sucrose synthesis by the *OsmK* kinase, which in turn is upregulated by the expected water deficit in the afternoon by *CaK* kinase (figure 1, and electronic supplementary material, figure S3a). A minor deviation can be observed (figure 2*b*) in that the increase in sucrose levels after dawn is faster in the model compared with the data. This might be related to transient stimulation of water and sucrose transport and hence consumption of sucrose in the morning due to opening of stomata pores, which was not considered in our model. The model also reproduces the experimentally observed gradual increase of *T6P* during the day (figure 2*c*,*d*, [39]). The detailed kinetics of *T6P* includes two phases of accumulation, a first minor peak after dawn and second major peak at dusk (figure 2*d*). This feature is not described because of insufficient knowledge of the molecular mechanisms. One possible explanation might be a time separation between transient transcriptional activation of TPS after dawn and the following post-translational regulation of TPS by *SnRK1*, however measurements of diurnal activity of TPS complexes are required to test this idea. The parallel accumulation of sucrose and *T6P* (figure 2*a*,*c*) agrees with the experimentally observed dependence of sucrose and *T6P* levels on the duration of photoperiod (electronic supplementary material, figure S7, [5]). This results in a strong correlation between end-of-the-day contents of sucrose and *T6P* under different photoperiods, in agreement with experimental observations (figure 2*e*, [45]). So far, no consistent explanation has been provided for this strong correlation of sucrose and T6P concentrations, which is persistently observed under various conditions [45]. In the context of our model, it can be explained by the cross-regulations between these two metabolites (figure 1), in which sucrose levels positively influence *T6P* levels (by the double negative regulation involving *SnRK1*) and conversely *T6P* levels positively affect sucrose levels by stimulating carbon partitioning towards sucrose synthesis in source tissues (involving *OsmK*). In summary, our model supports the hypothesis to explain the diurnal kinetics of sucrose and *T6P* by the regulation of their synthesis by *OsmK* and *SnRK1* kinases.

Both source and sink pools of *T6P* increase during the day in the simulations (figure 2*f*). The pools are regulated by local TPS activities, which are activated by light and inhibited by local *SnRK1*. The model predicts that sink *T6P* sharply increases at dusk due to inhibition of *SnRK1* by sink sugars, which exhibit high levels in the afternoon. Changes of *SnRK1* in source tissues are less pronounced and mainly caused by the increase of TPS abundance by light [60]. In summary, the model suggests that daily increase of sucrose due to expected water deficit results in accelerated accumulation of *T6P* in sink tissues, which upregulates sink demand for carbon in longer days as compared to short days (figure 2).

### 3.2. Diurnal regulation of starch degradation by the clock and sink's demand

**Figure 3.**
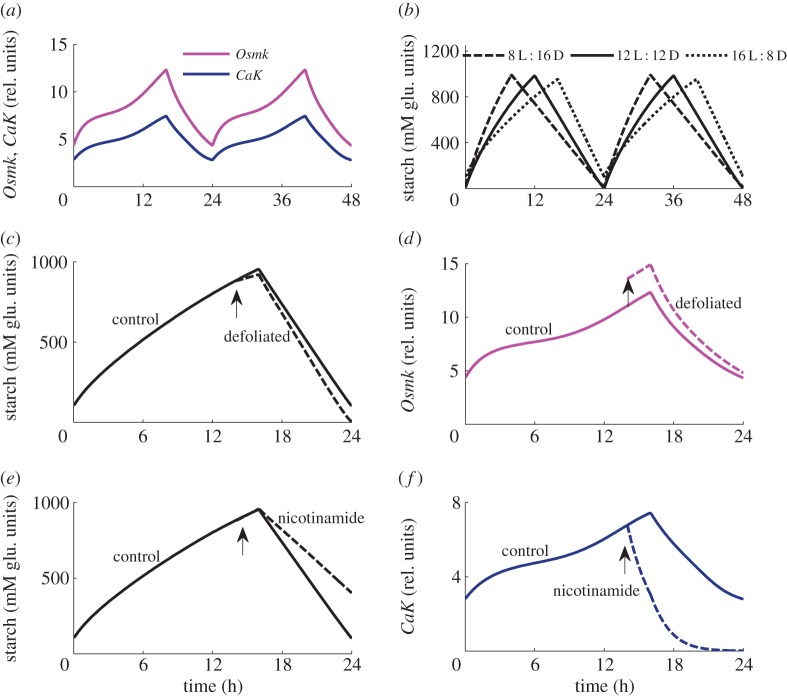
The proposed activation of starch degradation by *OsmK*. (*a*). Diurnal profiles of *OsmK* and its circadian component *CaK* in 16 L : 8 D are shown by magenta and blue lines, respectively. (*b*). Simulated diurnal starch profiles for plants grown in 8 L : 16 D, 12 L : 12 D and 16 L : 8 D, shown by dashed, solid and dotted lines, respectively. (*c*,*d*). Predicted changes in starch (*c*) and *OsmK* (*d*) kinetics in control (solid lines) and defoliated plants (dashed lines), in 16 L : 8 D. Defoliation was simulated by twofold decrease of the volume of source tissues 2 h before dusk (shown by arrow). Defoliation causes immediate increase of *OsmK* due to a relative increase of the sink's volume and hence sink demand. (*e*,*f*). Diurnal kinetics of starch (*e*) and *CaK* (*f*) in the control (solid lines) and nicotinamide-treated plants (dashed lines). The treatment by nicotinamide was simulated by switching off *CaK* activation (*k_sCaK_* = 0) 2 h before dusk (shown by arrow), which results in a decrease of *CaK* levels (*f*).

In our model, *OsmK* kinase leads to increased sucrose production also at night by accelerating starch degradation. Therefore, both the clock and sink demand affect starch degradation and hence the supply of carbon to sink tissues at night. As both circadian (*CaK*) and demand-related (*T6P*_si_) components of *OsmK* increase during the day (figures 3*a* and 2*d*), *OsmK* acts as a timer, which accelerates starch degradation in long days as compared to short days (figure 3*b*), which is in good agreement with experimental observations [3,4]. Although the necessity of the timer was proposed earlier [54], here we suggest *OsmK* kinase as the molecular candidate for the timer.

It remains to be resolved to what extent starch degradation is regulated on demand from sinks and how strong is the effect of the clock. We employed the model to suggest experiments, which may help to answer these open questions. The impact of demand by sinks is often manipulated by removing a part (e.g. half) of the mature leaves (defoliation), which increases the relative demand [13,61]. Such an experiment was already reported in 1984 [61]. The observation was that defoliation 1 h after dusk did not lead to an alteration of starch degradation rates. We explain this observation by the fact that at the time of defoliation, the maximal starch degradation rate is already set. Further, it suggests that other stresses resulting from the severe wounding inflicted by removing leaves do not seem to have an effect on starch degradation. This is indeed plausible, because the supply of carbon to the rest of the plant at night should be robust to similar interventions, including herbivore attacks. Thus, this experimental setting provides a useful instrument for the further exploration of the potential effect of sink demand on starch degradation. Therefore, we simulated different timings of defoliation, which, according to the model results, will provide a stronger insight into the regulatory mechanisms. When defoliation is applied 2 h before dusk to allow some time for changes of *OsmK* to occur (figure 3*c,d*), the model predicts that starch synthesis is decelerated immediately after defoliation, in agreement with experimental data [13]. The model explains this through the increased relative proportion of sink tissues and thus sink's demand in defoliated plants, which activates *OsmK* and shifts carbon partitioning towards sucrose synthesis. Further to this, the model predicts that starch degradation is slightly accelerated in defoliated plants (figure 3*c*) due to the increased *OsmK* (figure 3*d*).

Experimentally, the possible role of circadian release of Ca^2+^ (and hence *CaK*) in the regulation of starch breakdown can be tested by spraying plants with nicotinamide, which inhibits the biosynthesis of ADP-ribose, a metabolite necessary for the circadian release of Ca^2+^ in plants [28]. Figure 3*e* shows the result of a simulation of nicotinamide spraying 2 h before dusk, where the effect of nicotinamide was simulated by switching off *CaK* activation. The model predicts that reducing the *CaK* component of *OsmK* regulation (figure 3*a*,*f*) results in a measurable reduction of starch degradation (figure 3*e*). Thus, we predict that defoliation and nicotinamide spraying have opposite effects on starch degradation.

**Figure 4.**
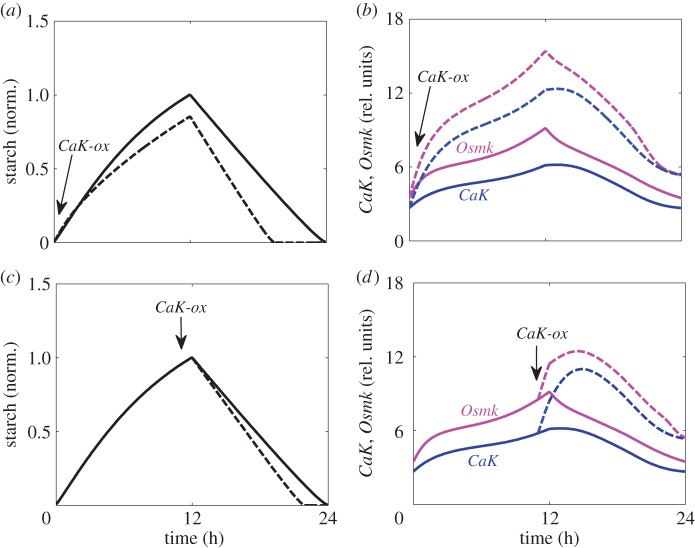
Predicted effects of inducible overexpression of *CaK* kinase on carbon fluxes. Simulated kinetics of starch (*a*,*c*) and *OsmK* and *CaK* (*b*,*d*) in 12 L : 12 D day cycle, after twofold induction of *CaK* at time 0 (*a*,*b*) or at time 11, 1 h before dusk (*c*,*d*). Solid and dashed lines correspond to control plants and plants after induction of *CaK*, which was simulated by increasing the rate of *CaK* activation twofold (parameter *k_sCaK_*). The arrow shows the time of induction. (Online version in colour.)

Simulation of an inducible overexpression of a Ca^2+^-dependent kinase (e.g. SnRK3.4, the robustly oscillating gene of the SnRK3 family) results in a particularly interesting model prediction, which is depicted in figure 4. Inducing *CaK* kinase at dawn (figure 4*a*,*b*) leads to an increase of *CaK* and *OsmK* levels (figure 4*b*), resulting in a shift of carbon partitioning towards sucrose synthesis and thus a decreased rate of starch synthesis (figure 4*a*). Concomitantly, higher *OsmK* levels are predicted to increase the starch degradation rate (figure 4*a*), which should lead to a period of carbon starvation before dawn. When applied shortly before dusk, *CaK* induction is predicted to result in a smaller, but notable acceleration of starch degradation (figure 4*c*,*d*). Our model predictions thus propose that nicotinamide spraying and *CaK* overexpression have opposite effects on starch degradation.

### 3.3. Regulation of sugar consumption in sink tissues by *SnRK1* and the clock

**Figure 5.**
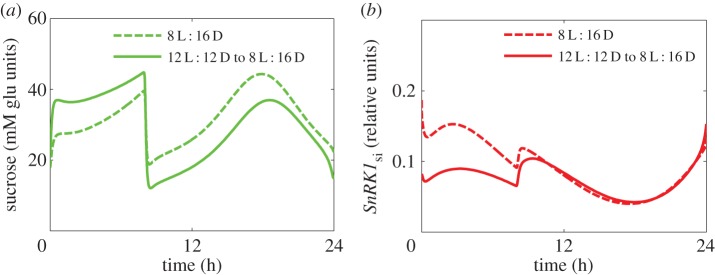
Inhibition of *SnRK1* by sugars in sink tissues helps plants to adjust sugar consumption to supply. Dynamics of the total sucrose level (*a*) and *SnRK1* activity in sink tissues (*b*) in simulations of plants transferred from 12 L : 12 D days to 8 L : 16 D days. The first and second days in 8 L : 16 D are shown by solid and dashed lines, respectively. (Online version in colour.)

In addition to regulation by the supply from the source tissues, sucrose levels are affected by the consumption of sucrose-derived hexose-phosphates in sink tissues. As in P2014, sugar consumption is inhibited by *SnRK1*. By contrast, in the present model, *SnRK1* activity in sink tissues is also inhibited by sugars, so that high sugar levels activate their own consumption through a double negative feed-forward loop (figure 1). Model simulations predict that the resulting effect on sucrose levels should be most pronounced when carbon supply is abruptly changing, as is for example the case in sudden light/dark transitions or severe herbivore attacks. Figure 5 illustrates this in a model simulation, in which *Arabidopsis* plants are transferred from normal to short days (early dusk). Normal day grown plants exhibit lower levels of *SnRK1* compared with short day grown plants (figure 5*b*, and electronic supplementary material, figure S3b,c), which results in a higher consumption rate and hence a more pronounced temporary reduction of sugars after the early dusk compared with short day grown plants (figure 5*a*). However, this rapid drop leads to an immediate release of *SnRK1* inhibition (figure 5*b*), which in turn slows sucrose consumption and thus prevents a further depletion of sugars. We conclude that the dynamic regulation of *SnRK1* by sugars is a key mechanism to avoid sugar depletion upon reduced carbon supply.

**Figure 6.**
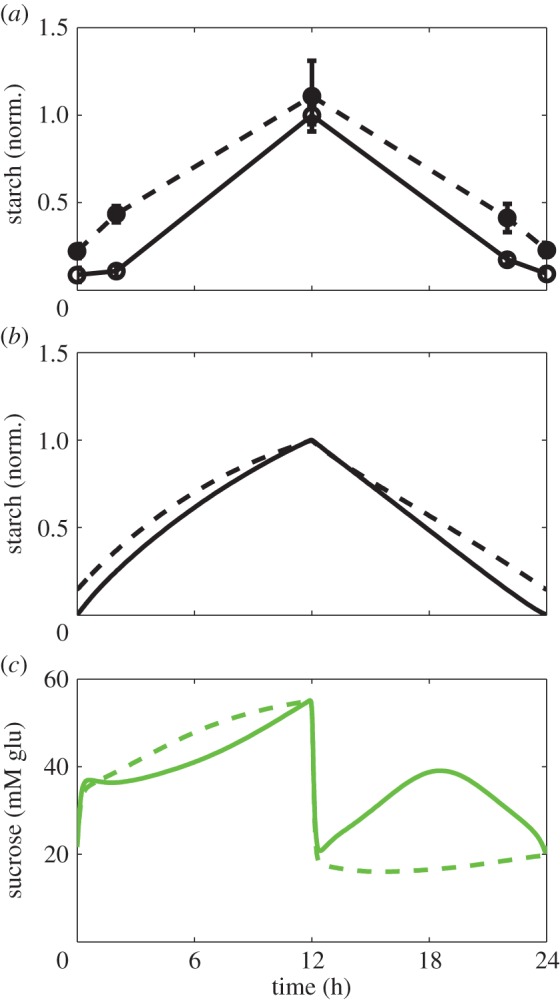
Diurnal changes in carbon metabolism in the clock mutant *elf3*. (*a*) Experimental data on starch kinetics in the wild-type and *elf3* mutant. Data points are redrawn from [65]. (*b*,*c*). Model simulations of starch (*b*) and sucrose (*c*) kinetics in the wild-type and *elf3* mutant. Experiments and simulations were performed for *Arabidopsis* plants grown in 12 L : 12 D. Solid lines correspond to the wild-type and dashed lines to the *elf3* mutant. The *elf3* mutant was simulated by setting the rate of ELF3 transcription (parameter *n*_3_ of the clock model) to zero. (Online version in colour.)

Sugar consumption is also regulated by the clock through activation by the morning complex LHY/CCA1 and inhibition by the EC (figure 1), which anticipate the diurnal increase and decrease of carbon supply (figure 2*a*,*b*). Including these circadian regulations improved the reproduction of sucrose dynamics compared with P2014 (figure 2*b*, and electronic supplementary material, figure S6a). In particular, the model can now explain the observed recovery of sucrose levels after the initial drop after dusk (figure 2*b*) through the circadian inhibition of sugar consumption by EC. To further explore this, we analysed the experimentally observed diurnal kinetics of starch and sucrose in the *elf3* mutant of the EC gene ELF3. This mutant displays a weak starch-excess phenotype, with a slightly slower starch degradation rate than the wild-type (figure 6*a*). Sucrose levels are slightly lower at night and higher during the day compared with the wild-type [65]. Figure 6*b*,*c* shows that our simulations of the *elf3* mutant qualitatively agree with experimental observations (figure 6*a*; [65]). By contrast, the P2014 model could neither describe the decreased starch degradation rate nor the changes in sucrose levels (electronic supplementary material, figure S6b,c). The proposed explanation of the phenotype of the *elf3* mutant is a perturbed balance between sugar supply and consumption. In particular, the missing inhibition of consumption by the EC at night results in low sugar levels and a concomitant activation of starvation mechanisms, which in turn downregulate β-amylase and thus starch degradation in the second part of the night (figure 6*b*). During the day, however, the model predicts the opposite changes: decreased LHY and CCA1 in the *elf3* mutant (electronic supplementary material, section E of S1) reduce sugar consumption, leading to their accumulation, which is also experimentally observed [65]. We conclude that the circadian regulation of sugar consumption optimizes accumulation of sucrose according to the daily fluctuations in carbon supply (figure 6*c*).

## 4. Conclusion

Our mathematical model describes the diurnal dynamics of carbohydrate storage and allocation in *Arabidopsis* plants. The model integrates metabolic and gene regulatory processes by including several mechanisms regulating carbon fluxes in source and sink tissues (figure 1). Sugar supply to growing sink tissues is regulated by environment-sensitive kinases, which in turn are under diurnal control of the circadian clock and reflect expected, regularly re-occurring water and carbon deficit, anticipated by the clock. Carbon deficit increases during the night and activates *SnRK1* kinase via its β subunit AKINβ1, inhibiting sucrose fluxes from source to sink. Water deficit increases during the day and activates *OsmK* kinase via its Ca^2+^-dependent component *CaK* kinase, which results in an activated sucrose flux during the day. Accumulation of sucrose during the day causes a parallel increase of *T6P*, a signalling metabolite which stimulates sink development and thus increases sink demand for carbon. This further activates *OsmK* kinase during the day in a positive feedback loop (figure 1). The model explains the experimentally observed parallel changes of sucrose and *T6P* levels [45,46] with the activation of *T6P* synthesis by sucrose, mediated by *SnRK1*. In summary, we suggest that environment-sensitive kinases activate accumulation of sucrose during the day and adjust plant development to the available sucrose.

During the night, sink tissues obtain carbon from degradation of stored starch. In our model, the increase of *OsmK* kinase during the day activates starch degradation the following night. This mechanism provides more carbon to actively developing sinks in long days. While we provide arguments based on experimental observations, which support our model assumptions about the regulatory mechanisms, these mechanisms remain hypothetical until they are confirmed experimentally. We therefore propose a number of new experiments, which allow validation of the model predictions which emerge as a consequence of the assumptions made. A key prediction of our model is that defoliation of plant leaves before dusk should lead to an accelerated starch degradation rate, because of the increased relative demand of carbon by sinks. We further predict that nicotinamide treatment before dusk will have the opposite effect and lead to a decreased starch degradation rate, because the circadian release of Ca^2+^ is inhibited. Moreover, starch degradation rate is also predicted to increase in experiments in which Ca^2+^-dependent kinases are overexpressed.

Because our model includes a detailed description of the circadian clock, it supports hypotheses by which mechanisms the clock regulates carbon fluxes in plants (figure 1). We propose three main mechanisms: (i) starch synthesis and carbon partitioning between starch and sucrose are regulated through the β subunit of *SnRK1*, AKINβ1; (ii) sink development and starch degradation is regulated by *CaK*, the Ca^2+^-dependent component of the osmotic kinase; (iii) the consumption of carbon by growing sinks is gated by the clock. Our simulations of the *elf3* mutant suggest that this circadian regulation of sugar consumption is important for plants to anticipate diurnal changes in sugar supply.

Our novel model components, which were all motivated by a number of experimental observations, now provide testable hypotheses regarding the molecular nature of key regulators of carbon metabolism in plants. We propose that the osmo-regulated kinase *OsmK* assumes the role of a timer, which is used to determine starch degradation rate as a function of the duration of the day. Further, we hypothesize that *T6P* serves as a signalling molecule to inform sugar-supplying organs about the demand by developing sink organs. Moreover, we propose a series of experiments, which have the potential to test some key aspects of our hypotheses. Whereas our theoretically derived hypotheses are of course not yet experimentally validated, we expect that our model can, already at this unconfirmed stage, serve as a valuable tool to further explore the regulatory principles of plant carbon metabolism guiding the coordination of supply and demand and the communication between source and sink tissues.

## Supplementary Material

Text S1

## Supplementary Material

Text S2

## Supplementary Material

Table S1
