# Supplementary material for: Mathematical modelling of diurnal regulation of carbohydrate allocation by osmo-related processes in plants
Source: J R Soc Interface. 2015 Mar 6;12(104):20141357. doi: 10.1098/rsif.2014.1357 (PMC4345503; doi:10.1098/rsif.2014.1357)
Supplement: Text S1 [file rsif20141357supp1.doc]

***Supplementary Information***

**Mathematical modelling of diurnal regulation of carbohydrate allocation by osmo-related processes in plants**

Alexandra Pokhilko, Oliver Ebenhöh

*Model description*

The model consists of six blocks of reactions, described in corresponding sections below: A - carbon fixation and starch synthesis; B - starch degradation; C - sucrose synthesis; D - consumption of sucrose by growing sinks; E - circadian clock and F – diurnal and circadian regulation of carbon metabolism (Fig.S1, Fig.1). Blocks A-E were mainly developed for the P2014 model , while block F describing diurnal regulation of carbon metabolism is new. The values of the model parameters are presented in Table S1.

1. *Carbon fixation and starch synthesis*

Carbon fixation in chloroplasts of source tissues is described based on the stoichiometry of the Calvin-Benson cycle (CBC) . It allows to relate the rates of carbon fixation () and starch synthesis ($v_{St\_source}$) with four pools of stromal phosphate groups: *THP_s_* - triose phosphates (PGA, GAP, DHAP) plus F16BP; *HP_s_* - hexose-phosphates (F6P, G6P, G1P) plus AGPG, *Pi_s_* - inorganic phosphate and *SP_s_* - other sugar-phosphates within the CBC (including RuBP), described by the following ordinary differential equations (ODEs, enumerated) and a conservation equation for the total amount of phosphate in the stroma (*Pi_tot_*):

$\frac{dTHP_{s}}{dt}=v_{SP\to TP}-v_{TP\to SP}-2v_{TP\to HP}-v_{TPT}\cdot\frac{v_{c}}{v_{s}}$ 1)

$\frac{dHP_{s}}{dt}=v_{TP\to HP}-v_{St\_source}-v_{HP\to SP}+v_{GPT2}\cdot\frac{v_{c}}{v_{s}}$ (2)

$\frac{dPi_{s}}{dt}=v_{TPT}\cdot\frac{v_{c}}{v_{s}}+v_{HP\to St}+v_{TP\to HP}-v_{Pi\to CBC}-v_{GPT2}\cdot\frac{v_{c}}{v_{s}}$ (3)

$$THP_{s}+HP_{s}+Pi_{s}+SP_{s}=Pi_{tot}$$

All variables are expressed in mM of phosphate units.

$v_{SP\to TP}$ is the rate of triose-phosphate formation in the RuBisCO carboxylase reaction and $v_{TP\to SP},v_{HP\to SP},v_{Pi\to CBC}$ are the consumption rates for THP, HP and Pi in the CBC during replenishment of SP, expressed as:

$$v_{SP\to TP}=3v_{phsyn}\cdot\frac{SP_{s}}{SP_{s}+K_{M\_SP}}$$

$$v_{TP\to SP}=\frac{3v_{phsyn}}{2}\cdot\frac{THP_{s}}{THP_{s}+K_{M\_THP}}$$

$$v_{HP\to SP}=\frac{v_{phsyn}}{2}\cdot\frac{HP_{s}}{HP_{s}+K_{M\_HP}}$$

$$v_{Pi\to CBC}=v_{phsyn}\cdot\frac{Pi_{s}}{Pi_{s}+K_{M\_ATPsynt}}$$

The rates of reactions conducted by stromal FBPase, sFBPase ($v_{TP\to HP}$) and AGPase ($v_{St\_source}$) are:

$$v_{TP\to HP}=V_{m\_sFBP}\cdot\frac{FBP_{s}}{FBP_{s}+K_{M\_sFBP}(1+F6P_{s}/K_{iF6P\_FBPase})}$$

$$v_{St\_source}=\frac{V_{m\_agp}\cdot L(t)\cdot G1P_{s}\cdot ATP_{s}}{(G1P_{s}+K_{M\_agp\_G1P})\cdot(ATP_{s}+K_{M\_agp\_ATP}(1+\frac{Pi_{s}}{K_{a\_agp\_PGA}(PGA_{s}+p_{0})}))}\cdot\frac{1}{1+(St/K_{i\_St})^{2}}$$

The last term in AGPase rate corresponds to chloroplast limits of starch (*St*) accumulation, with starch kinetics described by equation (30) of section F.

The export of triose-phosphates by triose-phosphate translocator (TPT), which exchanges triose-phosphates with inorganic phosphate (Pi) between stroma and cytosol is described as:

$$v_{TPT}=v_{TPT\_DHAP}+v_{TPT\_PGA}$$

$$v_{TPT\_DHAP}=V_{M\_TPT}(DHAP_{s}\cdot Pi_{c}-DHAP_{c}\cdot Pi_{s})$$

$$v_{TPT\_PGA}=V_{M\_TPT}(PGA_{s}\cdot Pi_{c}-PGA_{c}\cdot Pi_{s})$$

The rate of TPT is expressed in cytoplasmic concentration units, so an additional volume conversion coefficient $v_{c}/v_{s}$ (the ratio of cytoplasmic versus stromal volume) is present in the above equations (1)-(3).

The rate of GPT2 translocator ($v_{GPT2}$) exchanging hexose-phosphates with Pi, which is induced in the chloroplast membrane under stress conditions, is described in parts C below.

The rate of carbon fixation depends on presence of light:

$v_{phsyn}=v_{phsyn}^{obs}\cdot L(t)$,

where *L(t)* is a light function described in section F (*L(t)=1* when light is on and 0 when it is off) and is a parameter, which reflects the experimentally observed photosynthesis rate, which depends on experimental conditions, such as light intensity . In our model we used value, which corresponds to a moderate light intensity of 150 µmol/m^2^/s routinely used in labs .

All components of the *THP_s_* pool were expressed through *DHAP_s_* based on the rapid equilibrium assumption for intermediate enzymes :

$$GAP_{s}=DHAP_{s}/K_{eq\_TPiso}$$

$$FBP_{s}=K_{eq\_sAld}\cdot DHAP_{s}^{2}/K_{eq\_TPiso}$$

$$PGA_{s}=\frac{DHAP_{s}\cdot NADP\cdot Pi_{s}\cdot ADP_{s}}{K_{eq\_PGA\_GAP}\cdot K_{eq\_TPiso}\cdot ATP_{s}\cdot NADPH}$$

Where *DHAP_s_* is expressed through *THP_s_* as:

$$DHAP_{s}=\frac{\sqrt{b_{s}^{2}+4a\cdot THP_{s}}-b_{s}}{2a}$$

$$b_{s}=1+\frac{1}{K_{eq\_TPiso}}+\frac{NADP\cdot Pi_{s}\cdot ADP_{s}}{K_{eq\_PGA\_GAP}\cdot K_{eq\_TPiso}\cdot ATP_{s}\cdot NADPH}$$

$$a=\frac{2K_{eq\_sAld}}{K_{eq\_TPiso}}$$

Similarly, the components of the HP pool were determined using rapid equilibrium assumptions for the intermediate enzymes :

$$G6P_{s}=HP_{s}/(1+K_{eq\_PGM}+1/K_{eq\_PGI})$$

$$F6P_{s}=HP_{s}/(1+K_{eq\_PGI}+K_{eq\_PGI}\cdot K_{eq\_PGM})$$

$$G1P_{s}=HP_{s}\cdot K_{eq\_PGM}/(1+K_{eq\_PGM}+1/K_{eq\_PGI})$$

The parameters of carbon fixation and starch synthesis reactions are presented in Table S1 A.

1. *Starch degradation*

The block of reactions of starch degradation was developed in , based on published models . The final equation for the diurnal kinetics of starch is presented in the section F. It includes starch synthesis in presence of light and its degradation in darkness, which is modulated by diurnal regulators. The concentrations of starch and all intermediate forms of sugars produced during degradation of starch (glucose (G), maltose (M), maltotriose (G3), maltopentaose (G5) and starch linkage groups (Stlg) released by ISA) are expressed in glucosyl units. Starch is initially degraded by β amylase into maltose ($v_{St,\beta am}^{M}$) or maltotriose ($v_{St,\beta am}^{G3}$). In parallel ISA degrades starch into Stlg ($v_{St,isa}$). Stlg is degraded by β amylase to produce maltose ($v_{St\lg,\beta am}^{M}$) and maltotriose ($v_{St\lg,\beta am}^{G3}$). Maltotriose enters the disproportionation reaction catalysed by DPE1 (*v_dpe1_*), releasing glucose and maltopentaose, which is further degraded by β amylase ($v_{G5,\beta am}$). The final products of starch degradation, maltose and glucose, are exported from the chloroplast by MEX1 and GLUT translocators to the cytosol (*v_mex_* and *v_glut_*), where maltose is further converted to glucose and G1P by the coordinated action of DPE2 and PHS enzymes, described in one step (*vdpe2_phs*). Finally glucose is phosphorylated by hexokinase (*v_hxk_* ) and both G6P and G1P enter sucrose synthesis reactions, described in the next section. Additionally we assumed in the current model that β amylase reactions slow down under carbon starvation, as described in the main text. This was done by multiplying of the rates of β amylase reactions to *I_starv_*, described by equation (23) of section F. *I_starv_* is equal to 1 under normal conditions, but falls down under starvation. The equations describing the kinetics of starch degradation are presented below:

$$St_{\beta am}=f\cdot St;\text{ }St_{isa}=(1-f)\cdot St$$

$$v_{St,isa}=k_{isa}\cdot(1-L(t))\cdot V_{M\_isa}\cdot\frac{St_{isa}}{St_{isa}+K_{Misa}}$$

$$v_{St,\beta am}^{M}=\frac{k_{\beta}\cdot(1-L(t))\cdot I_{starv}\cdot V_{M\_\beta}\cdot f_{M}\cdot St_{\beta am}}{f_{M}\cdot St_{\beta am}+K_{M\beta}^{St}(1+\frac{M_{s}^{2}+M_{s}\cdot G3}{K_{Mr}})}$$

$$v_{St,\beta am}^{G3}=\frac{k_{\beta}\cdot(1-L(t))\cdot I_{starv}\cdot V_{M\_\beta}\cdot f_{G3}\cdot St_{\beta am}}{f_{G3}\cdot St_{\beta am}+K_{M\beta}^{St}(1+\frac{M_{s}^{2}+M_{s}\cdot G3}{K_{Mr}})}$$

$$v_{St\lg,\beta am}^{M}=\frac{k_{\beta}^{G}\cdot I_{starv}\cdot V_{M\_\beta}\cdot f_{M}\cdot\text{Stlg}}{f_{M}\cdot\text{Stlg}+K_{M\beta}^{G}(1+\frac{M_{s}^{2}+M_{s}\cdot G3}{K_{Mr}})}$$

$$v_{St\lg,\beta am}^{G3}=\frac{k_{\beta}^{G}\cdot I_{starv}\cdot V_{M\_\beta}\cdot f_{G3}\cdot\text{Stlg}}{f_{G3}\cdot\text{Stlg}+K_{M\beta}^{G}(1+\frac{M_{s}^{2}+M_{s}\cdot G3}{K_{Mr}})}$$

$$v_{G5,\beta am}=\frac{k_{\beta}^{G}\cdot I_{starv}\cdot V_{M\_\beta}\cdot G5}{G5+K_{M\beta}^{G}(1+\frac{M_{s}^{2}+M_{s}\cdot G3}{K_{Mr}})}$$

$$v_{dpe1}=V_{M\_dpe1}\cdot((G3/3)^{2}-\frac{(G5/5)\cdot G_{s}}{K_{eq}^{dpe}})$$

$$v_{dpe2\_phs}=V_{M\_dpe2}\cdot(M_{c}/2\cdot Pi_{c}-\frac{G1P_{c}\cdot G_{c}}{K_{eq}^{dpe2\_phs}})$$

$$v_{mex}=V_{M\_mex}\cdot(\frac{M_{s}}{M_{s}+K_{M\_mex}}-\frac{M_{c}}{M_{c}+K_{M\_mex}})$$

$$v_{glut}=V_{M\_glut}\cdot(\frac{G_{s}}{G_{s}+K_{M\_glut}}-\frac{G_{c}}{G_{c}+K_{M\_glut}})$$

$$v_{hxk}=V_{M\_hxk}\cdot\frac{G_{c}}{G_{c}+K_{M\_hxk}\cdot(1+G6P_{c}/K_{iG6P})}$$

$$k_{isa}=g(\frac{St_{isa\_\mathrm{int}}}{St_{tot\_\mathrm{int}}\cdot(1-f)+s_{0}})$$

$$k_{\beta}=g(\frac{St_{\beta am\_\mathrm{int}}}{St_{tot\_\mathrm{int}}\cdot f+s_{0}})$$

$g(x)=0.5-a\tan(10\cdot(x-1))/\pi$

$St_{tot\_\mathrm{int}}=St_{\beta am\_\mathrm{int}}+St_{isa\_\mathrm{int}}$

$\frac{dSt_{isa\_\mathrm{int}}}{dt}=v_{St,isa}\cdot X-k_{d\_\mathrm{int}}\cdot L(t)\cdot St_{isa\_\mathrm{int}}$ (4)

$\frac{dSt_{\beta am\_\mathrm{int}}}{dt}=v_{St,\beta am}^{M}\cdot X+v_{St,\beta am}^{G3}\cdot X-k_{d\_\mathrm{int}}\cdot L(t)\cdot St_{\beta am\_\mathrm{int}}$ (5)

$\frac{d\text{Stlg}}{dt}=v_{St,isa}\cdot X-v_{\text{Stlg,}\beta\text{am}}^{M}-v_{\text{Stlg,}\beta\text{am}}^{G3}$ (6)

$\frac{d\text{M}_{s}}{dt}=v_{\text{St,}\beta\text{am}}^{M}\cdot X+v_{\text{Stlg,}\beta\text{am}}^{M}+2/5\cdot v_{G5,\beta am}-v_{mex}\cdot v_{c}/v_{s}$ (7)

$\frac{dG3}{dt}=v_{\text{St,}\beta\text{am}}^{G3}\cdot X+v_{\text{Stlg,}\beta\text{am}}^{G3}+3/5\cdot v_{G5,\beta am}-6v_{dpe1}$ (8)

$\frac{dG_{s}}{dt}=v_{dpe1}-v_{glut}\cdot v_{c}/v_{s}$ (9)

$\frac{dG5}{dt}=5v_{dpe1}-v_{G5,\beta am}$ (10)

$\frac{dG_{c}}{dt}=v_{glut}+v_{dpe2\_phs}-v_{hxk}$ (11)

$\frac{dM_{c}}{dt}=v_{mex}-2v_{dpe2\_phs}$ (12)

The indices c/s in G and M correspond to cytoplasmic/stromal concentrations of glucose and maltose respectively; is the ratio of cytoplasmic-to-stromal volumes; *f* and (1-*f*) are fractions of starch degradable by β amylase and ISA and $f_{M}$ and are fractions of starch degraded to maltose and maltotriose; *L(t)* is the light function. *X* is a diurnal regulator of starch degradation (equation (28) of section F), which modulates the activities of the enzymes, bound to starch granules (β amylase and ISA). *St* is the total amount of starch; $St_{\beta am}$ and $St_{isa}$ are starch accessible to β amylase and ISA; $St_{\beta am\_\mathrm{int}}$ and are the amount of starch, which was degraded during a night by β amylase and by iso-amylase respectively. These terms are used to describe the competition between β amylase and ISA for the starch. The coefficients in equations for starch intermediates reflect the conversion of glucosyl units to concentration units.

The parameters of the starch degradation reactions are presented in Table S1 B.

1. *Sucrose synthesis*

During daytime the synthesis of sucrose in source tissues (suc_so_) starts from conversion of cytosolic triose-phosphates into F1,6P by aldolase (Fig. S1). F1,6P is next used for production of F6P in an irreversible reaction catalysed by cytosolic FBPase (cFBPase, $v_{cFBPase}$) . F6P enters a couple of reversible reactions catalysed by phosphoglucoisomerase PGI ($v_{PGI}$), phosphoglucomutase PGM ($v_{PGM}$) and UGPase ($v_{UGPase}$). The final step of sucrose synthesis is mediated by irreversible reaction of sucrose-phosphate synthase (SPS) ($v_{sps}$). Both cFBPase and SPS are diurnally regulated in parallel . SPS is directly regulated by phosphorylation, while cFBPase activity modulated through allosteric inhibition by the specific effector F26P. F26P is synthesized via phosphorylation of F6P by F6PK ($v_{F6PK}$), and F26P is dephosphorylated back to F6P by F26PP ($v_{F26PP}$). In our model cFBPase is diurnally regulated via phosphorylation of F26PP . The diurnal regulation of F26PP and SPS is described in the model through multiplying the rates of these enzymes to the function *_fdiurn_(t),* described in section F. In the night G6P and G1P, derived from starch (previous section), bypass cFBPase and enter the same set of reversible reactions as in the day (Fig. 1S), which leads to production of sucrose with only SPS controlling the flux at the end of the pathway . Cytosolic hexose-phosphates (F6P, G6P, G1P), UDPG, sucrose and F26P are presented in glucosyl units in our model. Cytosolic trioso-phosphates were expressed via the total pool of trioso-phosphates and F16P (*THP_c_*) similarly to the stromal *THP_s_* using rapid equilibrium approximation. The metabolites of *THP_c_* pool are presented in phosphate units in the model.

The reactions describing sucrose synthesis are presented below:

$\frac{dTHP_{c}}{dt}=v_{TPT}-2(v_{cFBPase}-v_{PFK})-v_{resp}$ (13)

$\frac{dF6P_{c}}{dt}=v_{cFBPase}-v_{PFK}+v_{PGI}-v_{sps}/2+v_{suc\_hydr\_so}/2$ (14)

$\frac{dG6P_{c}}{dt}=v_{HXK}+v_{PGM}-v_{PGI}-v_{GPT2}$ (15)

$\frac{dG1P_{c}}{dt}=v_{dpe2\_phs}-v_{PGM}-v_{UGPase}$ (16)

$\frac{dUDPG_{c}}{dt}=v_{UDPase}-v_{sps}/2+v_{suc\_hydr\_so}/2$ (17)

$\frac{dsuc_{so}}{dt}=v_{sps}-v_{suc\_hydr\_so}-v_{\exp}$ (18)

$\frac{dF26P_{c}}{dt}=v_{F6PK}-v_{F26PP}$ (19)

$$v_{PGI}=v_{M\_PGI}\cdot(G6P_{c}/K_{eq\_PGI}-F6P_{c})$$

$$v_{PGM}=v_{M\_PGM}\cdot(G1P_{c}/K_{eq\_PGM}-G6P_{c})$$

$$v_{UGPase}=v_{M\_UGPase}\cdot\frac{UTP_{c}\cdot G1P_{c}-UDPG_{c}\cdot PPi_{c}/K_{eq\_UGPase}}{UTP_{c}+K_{M\_UTP}}$$

$$v_{SPS}=v_{M\_SPS}\cdot f_{diurn}(t)\cdot\frac{F6P_{c}}{F6P_{c}+K_{M\_F6P}}\cdot\frac{UDPG_{c}}{UDPG_{c}+K_{M\_UDPG}}$$

$$v_{suc\_hydr\_so}=v_{M\_suc\_hydr\_so}\cdot suc_{so}$$

$$v_{cFBPase}=v_{M\_cFBPase}\cdot\frac{FBP_{c}}{FBP_{c}+K_{M\_cFBPase}(1+\frac{F6P_{c}}{K_{iF6P\_FBPase}}+\frac{F26P_{c}}{K_{iF26P}})}$$

$$v_{PFP}=v_{M\_PFP}\cdot(PPi_{c}\cdot F6P_{c}-Pi_{c}\cdot FBP_{c}/K_{eq\_PFP})$$

$$v_{F6PK}=v_{M\_F6PK}\cdot F6P_{c}/(F6P_{c}+K_{M\_F6PK}\cdot(1+PGA/K_{iPGA}))$$

$$v_{F26PP}=v_{M\_F26PP}\cdot f_{diurn}(t)\cdot\frac{F26P_{c}}{F26P_{c}+K_{M\_F26PP}(1+F6P_{c}/K_{iF6P\_F26PP})}$$

$$v_{resp}=v_{M\_resp}\cdot\frac{THP_{c}}{THP_{c}+K_{M\_resp}}$$

$$THP_{c}=PGA_{c}+GAP_{c}+DHAP_{c}+2FBP_{c}$$

$$GAP_{c}=DHAP_{c}/K_{eq\_TPiso}$$

$$FBP_{c}=K_{eq\_Ald}\cdot DHAP_{c}^{2}/K_{eq\_TPiso}$$

$$PGA_{c}=\frac{DHAP_{c}\cdot NADP\cdot Pi_{c}\cdot ADP_{c}}{K_{eq\_PGA\_GAP}\cdot K_{eq\_TPiso}\cdot ATP_{c}\cdot NADPH}$$

$$DHAP_{c}=\frac{\sqrt{b_{c}^{2}+4a\cdot THP_{c}}-b_{c}}{2a}$$

$$b_{c}=1+\frac{1}{K_{eq\_TPiso}}+\frac{NADP\cdot Pi_{c}\cdot ADP_{c}}{K_{eq\_PGA\_GAP}\cdot K_{eq\_TPiso}\cdot ATP_{c}\cdot NADPH}$$

$$a=\frac{2K_{eq\_Ald}}{K_{eq\_TPiso}}$$

$$v_{GPT2}=v_{M\_GPT2}\cdot GPT2(t)\cdot L(t)\cdot(G6P_{c}\cdot Pi_{s}-HP_{s}\cdot Pi_{c})$$

Where export of sucrose to sink tissues (*v_exp_*) described in the next section. Equations for *G6P_c_* and *HP_s_*, *Pi_s_* (part A above) additionally have a term describing GPT2 ($v_{GPT2}$), the G6P translocator, which exchanges G6P with Pi across the chloroplast membrane. GPT2 activity (*GPT2*) is assumed to be induced only in presence of light under limited carbon condition , as described by equation (29) of section F. Equations for hexokinase and DPE2 (producing *G6P_c_* and *G1P_c_*) are presented in part B above. In addition to reactions described in P2014, we added the hydrolysis of sucrose (*vsuc_hydr*), to account for the observed cycling of sucrose . The rate constant of the sucrose hydrolysis (*VM_suc_hydr_so*) was chosen to give the observed reversion of 25% of synthesised sucrose back to hexose-phosphate pool under normal conditions . The parameters of all reactions of sucrose synthesis are presented in Table S1 C.

1. *Consumption of sucrose by growing sinks*

Sucrose, which is synthesised in source tissues, is exported to sinks tissues ($v_{\exp}$), where it is converted to sugars and sugar-phosphates by action of sucrose – cleaving enzymes such as sucrose-synthase and invertase ($v_{suc\_hydr\_si}$). Similarly to P2014, sucrose metabolism in sinks is described in our model via changes in two variables: sucrose (*suc_si_* ) and a hexose/hexose-phosphate pool (*HP_si_*), both expressed in glucosyl units. $HP_{si}$ is consumed by various processes, mainly related with growth ($v_{cons}$) and for the synthesis of starch in sinks ($v_{St\_\sin k}$). Equation for $v_{St\_\sin k}$ is presented in section F. The equations describing sucrose metabolism in sink tissues are:

$\frac{dsuc_{si}}{dt}=v_{\exp}\cdot V_{so}/V_{si}-v_{suc\_hydr\_si}$ (20)

$\frac{dHP_{si}}{dt}=v_{suc\_hydr\_si}-v_{cons}-v_{St\_\sin k}$ (21)

$$v_{suc\_hydr\_si}=v_{M\_suc\_hydr\_si}\cdot suc_{si}-v_{M\_suc\_syn\_si}\cdot HP_{si}$$

$$v_{\exp}=v_{M\_\exp}\cdot(suc_{so}-suc_{si})$$

Here parameters $V_{so}$ and are the relative volumes of source and sink tissues, taking values between 0 and 1 both and the total volume ($V_{so}$ + ) is equal to 1 under normal conditions, but was changing in simulations of defoliation experiments presented in Results. The hydrolysis of sucrose is described as a balance between hydrolysis and synthesis processes. Since the relative impact of various enzymes in sucrose hydrolysis and synthesis in sink tissues on a whole plant level in unknown, we simply assumed that the hydrolysis rate is proportional to *suc_si_* and synthesis is proportional to *HP_si_*. The rate constants of sucrose hydrolysis and synthesis were chosen to correspond to the observed concentrations of sucrose and hexose-phosphates respectively . The rate of consumption of sugars $v_{cons}$ is diurnally regulated through inhibition by SnRK1_si_ and through the action of the clock as described in section F. The parameters of the consumption of sucrose by growing sinks are presented in Table S1 D together with parameters of diurnal regulation, described in section F.

1. *Modelling of the circadian clock in plants*

To describe the circadian regulation of *β*, *CaK* and sink’s consumption, the model was connected to the most recent version of the plant clock similarly to P2014. The clock structure consists of interlocked oscillators. The morning loop is based on the autoregulation of the key transcription factors *LHY* and *CCA1* through the activation of their own repressors - PRR proteins. The evening loop consists of EC (EVENING COMPLEX) genes *LUX*, *ELF3* and *ELF4*, which negatively regulate their own expression. The morning and evening loops are interlocked, in particular via inhibition of *PRR9* expression by the EC, which results in indirect stimulation of *LHY* and *CCA1* transcription by the EC, via double negative regulation . This explains the severe phenotype of the *elf3* mutant, which has no EC and additionally – low levels of LHY and CCA1 . Additionally to their interactions inside the clock, both LHY/CCA1 and EC complexes negatively regulate transcription of multiple target genes . The equation for LHY transcription was corrected similarly to to include the inhibition of acute LHY light response in LHY transcription by PRR proteins.

*F. Diurnal and circadian regulation of carbon metabolism*

In source tissues, photosynthesis provides carbon for sinks growth during daytime. In our model, carbon is fixed in chloroplasts in the Calvin-Benson cycle and resulting triose-phosphates (TP) are partitioned between starch synthesis pathway in chloroplasts and sucrose synthesis pathway in cytosol as described in sections A,C. Diurnal regulation of partitioning consists of parallel regulation of the key enzymes of sucrose synthesis pathway cFBPase and SPS (Fig.1S; through inhibition by SnRK1 kinase in source tissues (*SnRK1_so_*) similarly to P2014 and activation by osmo-sensitive kinase, called *OsmK* (Fig.1; . SPS is directly phosphorylated by kinases, while cFBPase is regulated via phosphorylation of fructose-6-phosphate phosphatase F26PP, which reduces the amount of fructose-2,6-phosphate (F26P) - an allosteric inhibitor of cFBPase (Fig.S1).

In our model *SnRK1* and *OsmK* kinase activities are regulated during the day accordingly to environmental conditions and circadian time (Fig.1). *SnRK1* is activated by total level of carbon deficit through its β subunit AKINβ1 (called *β* in the model), similarly to P2014. *β* is described as in P2014: It accumulates in darkness. Additionally it is stimulated by circadian complex LHY/CCA1 and inhibited by clock protein TOC1. Under carbon stress, *SnRK1* is stimulated by severe sugar drop, as observed in some mutants (e.g., in the *lhy/cca1* mutant) at the end of the night or in wild type plants during extended night . One possible mechanism might be related with activation of the βγ subunit of SnRK1 - AKINβγ . To describe this and other responses to night carbon stress (β-amylase, section B), we introduced into the model a sensor of low sugar levels during the night, *I_starv_* (from inhibitor of starvation). *I_starv_* is equal to 1 when sugar phosphates are in a normal physiological range and is reduced when hexose-phosphates in sinks (*HP_si_*) fall below a threshold level during the dark period. The decrease in *I_starv_* due to sugar drop activates *SnRK1* in our model. The equations for *SnRK1_so_, I_starv_, β* and the diurnal component of SPS and F26PP enzymes () are:

$\frac{dSnRK1_{so}}{dt}=k_{SnRK1}\cdot(\beta\cdot(1+\frac{1}{1+I_{starv}/K_{i\_Starv}})-SnRK1_{so})$ (22)

$\frac{dI_{starv}}{dt}=k_{Istarv}\cdot(L(t)+(1-L(t))\cdot\frac{HP_{si}^{3}}{HP_{si}^{3}+K_{starv}^{3}}-I_{starv})$ (23)

$\frac{d\beta}{dt}=k_{sb,1}\cdot\frac{K_{sb,1}^{2}}{TOC1^{2}+K_{sb,1}^{2}}+k_{sb,2}\cdot\frac{LHY^{2}}{LHY^{2}+K_{sb,2}^{2}}-k_{db}\cdot\beta\cdot L(t)$ (24)

$$f_{diurn}(t)=\frac{OsmK}{1+SnRK1_{so}/K_{i\_diurn}}$$

Where *L(t)* is a light function. *L(t)*=1 when light is present, 0 otherwise. The equation describing smooth transitions of the light function between light and dark is taken from our clock model :

$$\begin{matrix} L(t)=Loff+0.5\cdot Lamp\cdot(((1+\tanh((t-per\cdot floor(t/per)-dawn)/T))-(1+\tanh((t- \\ per\cdot floor(t/per)-dusk)/T))+(1+\tanh((t-per\cdot floor(t/per)-per)/T)))) \end{matrix}$$

Where *T* is twilight duration (we used *T*=0.05 h), *period* is the duration of the day (normally 24 h, except simulations of different T cycles), *dusk* and *dawn* are phases of dusk and dawn respectively (*dawn* normally equal to 0). tanh and floor – standard functions of hyperbolic tangent and rounding operation. Parameters *Loff* and *Lamp* are introduced in the clock model to simulate constant light and constant dark conditions. In diurnal cycle, which we used in all simulations presented in Results *Loff* =0 and *Lamp* =1. Photoperiod simulations were performed by varying the corresponding phase of dusk.

*OsmK* is activated by water deficit through its first component, called *CaK* in our model (from Ca^2+^ - dependent kinase; ). *CaK* increases during daytime due to inhibition by LHY/CCA1 and reduced degradation during daytime (Fig.1). This regulation provides a circadian peak in the afternoon, corresponding to the time of circadian release of Ca^2+^ and anticipated increase of water stress during the day . The decrease of *CaK* degradation during daytime is assumed to be gated by the clock via morning component PRR9, similarly to gating of α in P2014, to account for normal rate of starch degradation (and hence normal level of CaK) in experiments with skeleton photoperiods . The second component of *OsmK* is related with sink’s demand for carbon , which is assumed to be proportional to sink’s T6P (*T6P_si_*) (Fig.1; ). Regulation of T6P level is described below. The equations for *CaK* and *OsmK* are:

$\frac{dCaK}{dt}=k_{sCaK}\cdot(1+\frac{K_{iLHY}^{2}}{LHY^{2}+K_{iLHY}^{2}})-k_{dCaK}\cdot(1+\frac{1-L(t)}{1+(PRR9/K_{dCaK})^{2}})\cdot CaK$ (25)

$$OsmK=CaK+k_{OsmK}\cdot T6P_{si}\cdot V_{si}/(V_{si}+V_{so})$$

Where *V_si_* and *V_so_* are relative volumes of sink and source tissues, similar to P2014.

Sink tissues import sucrose and convert it to hexose-phosphates (*HP_si_*), which are consumed for growth (section D). The consumption of *HP_si_* is assumed to be diurnally regulated through inhibition by SnRK1 similarly to P2014 . In addition we added circadian regulation of the consumption via activation by LHY/CCA1 and inhibition by the evening complex (EC) (Fig.1), presumably mediated by growth stimulators PIF4 and PIF5 . Since SnRK1 is a global regulator of consumption according to the plant energy status, we assumed that the clock acts downstream of SnRK1. The rate of *HP_si_* consumption was described as:

$$v_{cons}=\frac{v_{M\_cons}\cdot HP_{si}}{1+(SnRK1_{si}\cdot(1+EC/K_{i\_consEC})/(K_{i\_consSnRK1}\cdot(1+LHY/K_{a\_consLHY})))^{2}}$$

The accumulation of T6P was assumed to be determined by TPS activity. The data suggests that the activity of the key enzyme of T6P synthesis TPS1 might be modulated through its binding to regulatory TPS proteins, mostly of class II , which are inhibited by SnRK1 . TPS proteins are additionally upregulated by light on transcriptional level . Therefore TPS activities in sink and source tissues were described as:

$\frac{TPS_{si}}{dt}=k_{aTPS1}+k_{aTPS2}\cdot L(t)-k_{dTPS}\cdot TPS_{si}\cdot(SnRK1_{si}+K_{iTPS})$ (26)

$\frac{TPS_{so}}{dt}=k_{aTPS1}+k_{aTPS2}\cdot L(t)-k_{dTPS}\cdot TPS_{so}\cdot(SnRK1_{so}+K_{iTPS})$ (27)

T6P levels were assumed in our model to be mainly determined by TPS activity. Therefore *T6P_si_* and *T6P_so_* were expressed as: *T6P_so_ = k_aT6P_ · TPS_so_ ; T6P_si_ = k_aT6P_ · TPS_si_.*

where *k_aT6P_* parameter relating T6P and TPS levels was varied to achieved correspondence with T6P data (Fig.2). The total levels of T6P (and similarly sucrose) in a whole plant were determined from the respective source and sink concentrations as: *T6P_tot_ = (T6P_si_ · V_si_ + T6P_so_ · V_so_)/( V_si_ + V_so_).*

*SnRK1_si_* was described similar to *SnRK1_so_*, but we increased *SnRK1_si_* activity compared to *SnRK1_so_* due to its activation by upstream kinases specific to sink tissues . Also we added inhibition of *SnRK1_si_* by *HP_si_* , which adjusts *SnRK1_si_* according to plant’s energy level. Besides we assumed that some part of SnRK1 activity is sequestered into TPS-related complexes, which is highly possible considering that both TPS and SnRK1 are capable to bind T6P . Since the molecular details of these interactions are not clear yet and concentration of TPS complexes is unknown (and thus dimensionless in our model), we simply expressed *SnRK1_si_* as:

$$SnRK1_{si}=\frac{k_{aSnRK1si}\cdot SnRK1_{so}}{1+HP_{si}/K_{i\_SnRK1\_HP}+TPS_{si}}$$

The partial downregulation of SnRK1 activity by high concentrations of TPS provides an additional decrease of *SnRK1_si_* in the afternoons of long days in our model, which provides good agreement with T6P data (Fig.2d).

Starch degradation in our model was set at dusk proportionally to starch level, similarly to P2014. This is supported by multiple experimental data, including the “early dusk” experiment, when starch degradation was instantly adjusted to the longer night when plants were subject to premature dusk , suggesting that starch degradation rate is set soon after dusk according to the starch level. The data also suggest that starch degradation is regulated by a timer accordingly to the time when dusk happens . In our model osmo-sensitive kinase *OsmK* was assumed to be that timer. *OsmK* increases during daytime and stimulates starch degradation, possibly via phosphorylation of related proteins . Thus, higher level of *OsmK* in long days increases the rate of starch degradation compared to shorter days, in agreement with data (Fig.4B; ). The data suggest that the rate of starch degradation is set soon after dusk , which is probably related to the formation of multi-protein starch-degrading enzymatic complexes soon after lights-off . The dusk setting of starch degradation rate was described similarly to P2014 via a variable *X*, which reflects amount of active enzymatic complexes on starch surface. *X* changes in the light according to levels of *OsmK* and starch, but remains constant in darkness:

$\frac{dX}{dt}=k_{sX}\cdot OsmK\cdot St\cdot L(t)-k_{dX}\cdot L(t)\cdot X$ (28)

Under carbon stress conditions starch synthesis might also increase through temporal induction of GPT2 translocator in the morning. GPT2 redirects a part of cytosolic hexose-phosphate pool back to chloroplasts . GPT2 was described similarly to P2014:

$\frac{dGPT2}{dt}=k_{sGPT}\cdot L(t)\cdot\frac{SnRK1_{so}^{4}}{SnRK1_{so}^{4}+K_{sGPT}^{4}}-k_{dGPT}\cdot GPT2$ (29)

The kinetics of starch accumulation was described was described as in P2014:

$\frac{St}{dt}=v_{St\_source}\cdot V_{so}+v_{St\_\sin k}\cdot V_{si}-(v_{St,\beta am}^{M}+v_{St,\beta am}^{G3}+v_{St,isa})\cdot X$ (30)

Were the rate of starch degradation was determined by the rates of β amylase and ISA (,$v_{St,\beta am}^{G3}$,$v_{St,isa}$) (section B). Starch degradation rate is diurnally modulated by the model component *X*, described above (eq.28). The level of β amylase was assumed to be constant in normal conditions, but downregulated under carbon stress via proportional dependence to the level of the inhibitor of starvation *I_starv_*, which is decreases upon drop of sugars, eq.23. The rate of starch synthesis is described after P2014:

$$v_{St\_source}=\frac{V_{m\_agp}\cdot L(t)\cdot G1P_{s}\cdot ATP_{s}}{(G1P_{s}+K_{M\_agp\_G1P})\cdot(ATP_{s}+K_{M\_agp\_ATP}(1+\frac{Pi_{s}}{K_{a\_agp\_PGA}PGA_{s}+p_{0}}))}\cdot\frac{1}{1+(St/K_{i\_St})^{2}}$$

$$v_{St\_\sin k}=(V_{M\_St\_\sin k}+V_{M\_GPT2}\cdot GPT2)\cdot L(t)\cdot HP_{si}\cdot\frac{1}{1+(St/K_{i\_St})^{2}}$$

Where *vSt_source* describes AGPase activity in source tissues and *vSt_sink* describes the rate of starch synthesis in sink tissues. The last term corresponds to chloroplast limits of starch accumulation.

All variables in this section (*CaK, Osm, β, SnRK1_so_, SnRK1_si_, I_starv_, X TPS_so_, TPS_si_, GPT2*) except T6P are dimensionless. T6P is expressed in µM. Hill coefficient 2 was used for the effects of clock components TOC1, LHY and PRR9 since it is known that these proteins work as dimers . Also we used Hill coefficient 2 for the effect of SnRK1 on sugar consumption because bZIP transcription factors downstream of SnRK1, which mediate SnRK responses act in dimer forms . For comparison with experimental data, the following volume coefficients were used: the stromal volume is assumed to be *v_s_* = 65 µl/g FW=65 µl/mg Chl, the cytosolic volume vc =23 µl/g FW=23 µl/mg Chl .

The schematic description of the differences between P2014 and the current model is presented in Fig. S2. Based on existing data we assumed that the same mechanism, mediated by *OsmK* kinase, regulates sucrose supply during day and night . Thus, in addition to activation of sucrose synthesis , *OsmK* activates starch degradation . Therefore, *OsmK* assumes the role of the hypothetical component α in P2014 (Fig. S2). In contrast to the unified mechanism of sucrose supply regulation in current model, P2014 had two different, non-related hypothetical mechanisms to activation sucrose supply during day (via *D*) and night (via α) (Fig. S2). *OsmK* integrates demand signals from the sinks and cues from the clock related to expected water deficit, anticipated by the Ca^2+^-dependent component of *OsmK* kinase (*CaK* kinase, ) (Fig. S2), This provides a mechanism for stimulation of sucrose synthesis and starch degradation by sink demand and the clock . The stimulation of starch degradation by sink demand is a new model property, which results in predictions for perturbations such as defoliation, on starch degradation, which can be tested experimentally (see Results). The second hypothetical component in P2014, the demand regulator D, was also replaced by a specific molecular candidate, namely the signaling metabolite *T6P* (Fig. S2). This proposition is based on a range of experimental data on the effects of *T6P_si_* on sucrose metabolism . To describe the observed diurnal regulation of *T6P_si_* levels , we included the regulation of *T6P* synthesis by *SnRK* kinase . A further improvement involves the compartmentalization of *SnRK1* activity. While in P2014 *SnRK1* (termed *I*) was assumed to change similarly in both source and sink tissues, we now take into account the observed inhibition of *SnRK1* by sugars specifically in sink tissues . Further, the current model was extended to include transcriptional upregulation of *SnRK1* levels by carbon starvation , which is observed in some mutants or under low light conditions. And finally, the current model was extended by including circadian regulation of sucrose consumption, which allows plants to anticipate diurnal changes in sugar supply (Fig.1, see Results).

Therefore we substantially rewired the scheme of diurnal regulators as depicted in Fig. S2. These modifications resulted in alteration (or addition) of equations for *OsmK,* *CaK, T6P, SnRK1, f_diurn_(t)* and *v_cons_*. 15 parameters in P2014 (in the equations for *α,* *D, SnRK1, f_diurn_(t)* and *v_cons_*) were replaced by 19 parameters reflecting the changes described above. All other parameters are conserved between the two models. Similarly to P2014, we fitted the parameters of diurnal regulation to the data on the kinetics of the system under different photoperiods. Fig. S4a-d and Fig.2b,d,e demonstrate that our model provides good agreement with the existing data on starch, sucrose and *T6P* timecourses under various photoperiods. Our simulations also showed that the model retains all other properties of P2014 and allows describing the response of starch kinetics to various genetic and environmental perturbations. In particular, Figs. S4e,f show that the model corresponds to the data on immediate adjustment of starch degradation rate to unexpected early dusk. The model also corresponds to the data on the increase of starch levels in starch-excess mutants such as *lsf1* mutant (Fig. S4g,h). Similarly to P2014, our model correctly describes changes in starch kinetics upon various perturbations of the normal light cycle and the circadian clock, such as skeleton photoperiods, night pulse, different T cycles (duration of the day) and clock mutants *lhy/cca1, prr7/prr9, toc1* (Fig. S5). In addition to retaining the properties of P2014, our current model allows describing the diurnal kinetics of *T6P*, an important regulator of plant development (Fig.2). The description of sucrose kinetics was improved compared to P2014 (Fig. S6a versus Fig. 2b). Now the model describes important features of the sucrose dynamics, such as its accumulation during the day and partial restoration of sucrose levels in the night, after the transient decrease after dusk (Fig.2b). The model explains sucrose accumulation in the day by a stimulation of sucrose synthesis by *OsmK* in the afternoon and circadian upregulation of sucrose consumption in the morning. Night restoration of sucrose levels is explained by a circadian inhibition of sucrose consumption at night (Fig 6, see Results for more details). The inclusion of the circadian regulation of sugar consumption by the clock also improved the description of starch and sucrose dynamics in the *elf3* mutant (Fig. 6, Fig. S6b,c). Thus the new model structure allowed us to quantitatively describe substantially more data compared to P2014, while providing an equally good agreement with the data, which could be described before. Besides, the inclusion of the specific molecular candidates as diurnal regulators of carbon metabolism allows for experimentally testable predictions how various perturbations will affect carbon allocation and fluxes (see Results).

**References**

1. Pokhilko A, Flis A, Sulpice R, Stitt M, Ebenhoh O. Adjustment of carbon fluxes to light conditions regulates the daily turnover of starch in plants: a computational model. Molecular bioSystems. 2014;10(3):613-27.

2. Farquhar GD, von Caemmerer S, Berry JA. A biochemical model of photosynthetic CO2 assimilation in leaves of C3 species. Planta. 1980;149:78-90.

3. Pyl ET, Piques M, Ivakov A, Schulze W, Ishihara H, Stitt M, et al. Metabolism and growth in Arabidopsis depend on the daytime temperature but are temperature-compensated against cool nights. Plant Cell. 2012;24(6):2443-69.

4. Strand A, Zrenner R, Trevanion S, Stitt M, Gustafsson P, Gardestrom P. Decreased expression of two key enzymes in the sucrose biosynthesis pathway, cytosolic fructose-1,6-bisphosphatase and sucrose phosphate synthase, has remarkably different consequences for photosynthetic carbon metabolism in transgenic Arabidopsis thaliana. Plant J. 2000;23(6):759-70.

5. Arrivault S, Guenther M, Ivakov A, Feil R, Vosloh D, van Dongen JT, et al. Use of reverse-phase liquid chromatography, linked to tandem mass spectrometry, to profile the Calvin cycle and other metabolic intermediates in Arabidopsis rosettes at different carbon dioxide concentrations. Plant J. 2009;59(5):826-39.

6. Pettersson G, Ryde-Pettersson U. A mathematical model of the Calvin photosynthesis cycle. European journal of biochemistry / FEBS. 1988;175(3):661-72.

7. Zhu XG, de Sturler E, Long SP. Optimizing the distribution of resources between enzymes of carbon metabolism can dramatically increase photosynthetic rate: a numerical simulation using an evolutionary algorithm. Plant physiology. 2007;145(2):513-26.

8. Nag A, Lunacek M, Graf PA, Chang CH. Kinetic modeling and exploratory numerical simulation of chloroplastic starch degradation. BMC systems biology. 2011;5:94.

9. Shiraishi F, Kawakami K, Yuasa A, Kojima T, Kusunoki K. Kinetic expression for maltose production from soluble starch by simultaneous use of beta-amylase and debranching enzymes. Biotechnol Bioeng. 1987;30(3):374-80.

10. Stitt MH, S; Kerr, P. Control of photosynthetic sucrose synthesis. In: Hatch MD, Boardman NK, editors. The Biochemistry of Plants. 10. New York: Academic Press; 1987. p. 327–409.

11. Quick WP, Schaffer AA. Sucrose metabolism in sources and sinks. In: Zamski E, Schaffer AA, editors. Photoassimilate distribution in plants and crops: Source-sink relationships. New York: Marcel Dekker; 1996. p. 115-56.

12. MacRae E, Lunn JE. Control of sucrose biosynthesis. In: Plaxton WC, McManus MT, editors. Control of primary metabolism in plants. Annual plant reviews. 22. Oxford: Blackwell Publishing Ltd.; 2006.

13. Kunz HH, Hausler RE, Fettke J, Herbst K, Niewiadomski P, Gierth M, et al. The role of plastidial glucose-6-phosphate/phosphate translocators in vegetative tissues of Arabidopsis thaliana mutants impaired in starch biosynthesis. Plant Biol (Stuttg). 2010;12 Suppl 1:115-28.

14. Nagele T, Henkel S, Hormiller I, Sauter T, Sawodny O, Ederer M, et al. Mathematical modeling of the central carbohydrate metabolism in Arabidopsis reveals a substantial regulatory influence of vacuolar invertase on whole plant carbon metabolism. Plant physiology. 2010;153(1):260-72.

15. Sulpice R, Flis A, Ivakov AA, Apelt F, Krohn N, Encke B, et al. Arabidopsis coordinates the diurnal regulation of carbon allocation and growth across a wide range of photoperiods. Molecular plant. 2014;7(1):137-55.

16. Nielsen TH, Veierskov B. Regulation of Carbon Partitioning in Source and Sink Leaf Parts in Sweet Pepper (Capsicum annuum L.) Plants : Role of Fructose 2,6-Bisphosphate. Plant physiology. 1990;93(2):637-41.

17. Pokhilko A, Mas P, Millar AJ. Modelling the widespread effects of TOC1 signalling on the plant circadian clock and its outputs. BMC systems biology. 2013;7:23.

18. Pokhilko A, Fernandez AP, Edwards KD, Southern MM, Halliday KJ, Millar AJ. The clock gene circuit in Arabidopsis includes a repressilator with additional feedback loops. Mol Syst Biol. 2012;8:574-87.

19. Dixon LE, Knox K, Kozma-Bognar L, Southern MM, Pokhilko A, Millar AJ. Temporal repression of core circadian genes is mediated through EARLY FLOWERING 3 in Arabidopsis. Curr Biol. 2011;21(2):120-5.

20. Seaton DD, Ebenhoh O, Millar AJ, Pokhilko A. Regulatory principles and experimental approaches to the circadian control of starch turnover. Journal of the Royal Society, Interface / the Royal Society. 2014;11(91):20130979.

21. Stitt M, Lunn J, Usadel B. Arabidopsis and primary photosynthetic metabolism - more than the icing on the cake. Plant J. 2010;61(6):1067-91.

22. Rufty TW, Huber SC. Changes in Starch Formation and Activities of Sucrose Phosphate Synthase and Cytoplasmic Fructose-1,6-bisphosphatase in Response to Source-Sink Alterations. Plant physiology. 1983;72(2):474-80.

23. Sugden C, Donaghy PG, Halford NG, Hardie DG. Two SNF1-related protein kinases from spinach leaf phosphorylate and inactivate 3-hydroxy-3-methylglutaryl-coenzyme A reductase, nitrate reductase, and sucrose phosphate synthase in vitro. Plant physiology. 1999;120(1):257-74.

24. Kulma A, Villadsen D, Campbell DG, Meek SE, Harthill JE, Nielsen TH, et al. Phosphorylation and 14-3-3 binding of Arabidopsis 6-phosphofructo-2-kinase/fructose-2,6-bisphosphatase. Plant J. 2004;37(5):654-67.

25. Toroser D, Huber SC. Protein phosphorylation as a mechanism for osmotic-stress activation of sucrose-phosphate synthase in spinach leaves. Plant physiology. 1997;114(3):947-55.

26. Boudsocq M, Droillard MJ, Barbier-Brygoo H, Lauriere C. Different phosphorylation mechanisms are involved in the activation of sucrose non-fermenting 1 related protein kinases 2 by osmotic stresses and abscisic acid. Plant molecular biology. 2007;63(4):491-503.

27. Graf A, Schlereth A, Stitt M, Smith AM. Circadian control of carbohydrate availability for growth in Arabidopsis plants at night. Proceedings of the National Academy of Sciences of the United States of America. 2010;107(20):9458-63.

28. Gibon Y, Blasing OE, Palacios-Rojas N, Pankovic D, Hendriks JH, Fisahn J, et al. Adjustment of diurnal starch turnover to short days: depletion of sugar during the night leads to a temporary inhibition of carbohydrate utilization, accumulation of sugars and post-translational activation of ADP-glucose pyrophosphorylase in the following light period. Plant J. 2004;39(6):847-62.

29. Blasing OE, Gibon Y, Gunther M, Hohne M, Morcuende R, Osuna D, et al. Sugars and circadian regulation make major contributions to the global regulation of diurnal gene expression in Arabidopsis. Plant Cell. 2005;17(12):3257-81.

30. Gissot L, Polge C, Jossier M, Girin T, Bouly JP, Kreis M, et al. AKINbetagamma contributes to SnRK1 heterotrimeric complexes and interacts with two proteins implicated in plant pathogen resistance through its KIS/GBD sequence. Plant physiology. 2006;142(3):931-44.

31. Martinez-Barajas E, Delatte T, Schluepmann H, de Jong GJ, Somsen GW, Nunes C, et al. Wheat grain development is characterized by remarkable trehalose 6-phosphate accumulation pregrain filling: tissue distribution and relationship to SNF1-related protein kinase1 activity. Plant physiology. 2011;156(1):373-81.

32. Coello P, Hey SJ, Halford NG. The sucrose non-fermenting-1-related (SnRK) family of protein kinases: potential for manipulation to improve stress tolerance and increase yield. Journal of experimental botany. 2011;62(3):883-93.

33. Robertson FC, Skeffington AW, Gardner MJ, Webb AA. Interactions between circadian and hormonal signalling in plants. Plant molecular biology. 2009;69(4):419-27.

34. Johnson CH, Knight MR, Kondo T, Masson P, Sedbrook J, Haley A, et al. Circadian oscillations of cytosolic and chloroplastic free calcium in plants. Science. 1995;269(5232):1863-5.

35. Dodd AN, Gardner MJ, Hotta CT, Hubbard KE, Dalchau N, Love J, et al. The Arabidopsis circadian clock incorporates a cADPR-based feedback loop. Science. 2007;318(5857):1789-92.

36. Rufty TW, Huber SC, Kerr PS. Effects of canopy defoliation in the dark on the activity of sucrose phosphate synthase. Plant Science Letters. 1984;34:247-52.

37. van Dijken AJ, Schluepmann H, Smeekens SC. Arabidopsis trehalose-6-phosphate synthase 1 is essential for normal vegetative growth and transition to flowering. Plant physiology. 2004;135(2):969-77.

38. Wahl V, Ponnu J, Schlereth A, Arrivault S, Langenecker T, Franke A, et al. Regulation of flowering by trehalose-6-phosphate signaling in Arabidopsis thaliana. Science. 2013;339(6120):704-7.

39. Baena-Gonzalez E, Sheen J. Convergent energy and stress signaling. Trends in plant science. 2008;13(9):474-82.

40. Niwa Y, Yamashino T, Mizuno T. The circadian clock regulates the photoperiodic response of hypocotyl elongation through a coincidence mechanism in Arabidopsis thaliana. Plant & cell physiology. 2009;50(4):838-54.

41. Zang B, Li H, Li W, Deng XW, Wang X. Analysis of trehalose-6-phosphate synthase (TPS) gene family suggests the formation of TPS complexes in rice. Plant molecular biology. 2011;76(6):507-22.

42. Vandesteene L, Ramon M, Le Roy K, Van Dijck P, Rolland F. A single active trehalose-6-P synthase (TPS) and a family of putative regulatory TPS-like proteins in Arabidopsis. Molecular plant. 2010;3(2):406-19.

43. Glinski M, Weckwerth W. Differential multisite phosphorylation of the trehalose-6-phosphate synthase gene family in Arabidopsis thaliana: a mass spectrometry-based process for multiparallel peptide library phosphorylation analysis. Molecular & cellular proteomics : MCP. 2005;4(10):1614-25.

44. Harthill JE, Meek SE, Morrice N, Peggie MW, Borch J, Wong BH, et al. Phosphorylation and 14-3-3 binding of Arabidopsis trehalose-phosphate synthase 5 in response to 2-deoxyglucose. Plant J. 2006;47(2):211-23.

45. Gibon Y, Usadel B, Blaesing OE, Kamlage B, Hoehne M, Trethewey R, et al. Integration of metabolite with transcript and enzyme activity profiling during diurnal cycles in Arabidopsis rosettes. Genome biology. 2006;7(8):R76.

46. Schluepmann H, van Dijken A, Aghdasi M, Wobbes B, Paul M, Smeekens S. Trehalose mediated growth inhibition of Arabidopsis seedlings is due to trehalose-6-phosphate accumulation. Plant physiology. 2004;135(2):879-90.

47. Yadav UP, Ivakov A, Feil R, Duan GY, Walther D, Giavalisco P, et al. The sucrose-trehalose 6-phosphate (Tre6P) nexus: specificity and mechanisms of sucrose signalling by Tre6P. Journal of experimental botany. 2014;65(4):1051-68.

48. Shen W, Reyes MI, Hanley-Bowdoin L. Arabidopsis protein kinases GRIK1 and GRIK2 specifically activate SnRK1 by phosphorylating its activation loop. Plant physiology. 2009;150(2):996-1005.

49. Zhang Y, Primavesi LF, Jhurreea D, Andralojc PJ, Mitchell RA, Powers SJ, et al. Inhibition of SNF1-related protein kinase1 activity and regulation of metabolic pathways by trehalose-6-phosphate. Plant physiology. 2009;149(4):1860-71.

50. Toroser D, Plaut Z, Huber SC. Regulation of a plant SNF1-related protein kinase by glucose-6-phosphate. Plant physiology. 2000;123(1):403-12.

51. Nunes C, Primavesi LF, Patel MK, Martinez-Barajas E, Powers SJ, Sagar R, et al. Inhibition of SnRK1 by metabolites: tissue-dependent effects and cooperative inhibition by glucose 1-phosphate in combination with trehalose 6-phosphate. Plant physiology and biochemistry : PPB / Societe francaise de physiologie vegetale. 2013;63:89-98.

52. Graf A, Smith AM. Starch and the clock: the dark side of plant productivity. Trends in plant science. 2011;16(3):169-75.

53. Bayer RG, Stael S, Rocha AG, Mair A, Vothknecht UC, Teige M. Chloroplast-localized protein kinases: a step forward towards a complete inventory. Journal of experimental botany. 2012;63(4):1713-23.

54. Rocha AMG. Calcium regulation in chloroplasts and the role of calcium-dependent phosphorylation of transketolase in carbon metabolism: Ludwig-Maximilians-Universität München; 2012.

55. Stael S, Rocha AG, Wimberger T, Anrather D, Vothknecht UC, Teige M. Cross-talk between calcium signalling and protein phosphorylation at the thylakoid. Journal of experimental botany. 2012;63(4):1725-33.

56. Makhmoudova A, Williams D, Brewer D, Massey S, Patterson J, Silva A, et al. Identification of multiple phosphorylation sites on maize endosperm starch branching enzyme IIb, a key enzyme in amylopectin biosynthesis. The Journal of biological chemistry. 2014;289(13):9233-46.

57. Chigri F, Hormann F, Stamp A, Stammers DK, Bolter B, Soll J, et al. Calcium regulation of chloroplast protein translocation is mediated by calmodulin binding to Tic32. Proceedings of the National Academy of Sciences of the United States of America. 2006;103(43):16051-6.

58. Gibon Y, Pyl ET, Sulpice R, Lunn JE, Hohne M, Gunther M, et al. Adjustment of growth, starch turnover, protein content and central metabolism to a decrease of the carbon supply when Arabidopsis is grown in very short photoperiods. Plant, cell & environment. 2009;32(7):859-74.

59. Lu Y, Gehan JP, Sharkey TD. Daylength and circadian effects on starch degradation and maltose metabolism. Plant physiology. 2005;138(4):2280-91.

60. Streb S, Zeeman SC. Starch metabolism in Arabidopsis. Arabidopsis Book. 2012;10:e0160.

61. Stettler M, Eicke S, Mettler T, Messerli G, Hortensteiner S, Zeeman SC. Blocking the metabolism of starch breakdown products in Arabidopsis leaves triggers chloroplast degradation. Molecular plant. 2009;2(6):1233-46.

62. Usadel B, Blasing OE, Gibon Y, Poree F, Hohne M, Gunter M, et al. Multilevel genomic analysis of the response of transcripts, enzyme activities and metabolites in Arabidopsis rosettes to a progressive decrease of temperature in the non-freezing range. Plant, cell & environment. 2008;31(4):518-47.

63. O'Neill JS, van Ooijen G, Le Bihan T, Millar AJ. Circadian clock parameter measurement: characterization of clock transcription factors using surface plasmon resonance. J Biol Rhythms. 2011;26(2):91-8.

64. Fujiwara S, Wang L, Han L, Suh SS, Salome PA, McClung CR, et al. Post-translational regulation of the Arabidopsis circadian clock through selective proteolysis and phosphorylation of pseudo-response regulator proteins. The Journal of biological chemistry. 2008;283(34):23073-83.

65. Yakir E, Hilman D, Kron I, Hassidim M, Melamed-Book N, Green RM. Posttranslational regulation of CIRCADIAN CLOCK ASSOCIATED1 in the circadian oscillator of Arabidopsis. Plant physiology. 2009;150(2):844-57.

66. Jakoby M, Weisshaar B, Droge-Laser W, Vicente-Carbajosa J, Tiedemann J, Kroj T, et al. bZIP transcription factors in Arabidopsis. Trends in plant science. 2002;7(3):106-11.

67. Winter H, Robinson DG, Heldt HW. Subcellular volumes and metabolite concentrations in spinach leaves. Planta. 1996;193:530-5.

68. Zrenner R, Stitt M. Comparison of the effect of rapidly and gradually developing water-stress on carbohydrate metabolism in spinach leaves. Plant, Cell and Environment. 1991;14:939-46.

69. Robinson SP. Osmotic adjustment by intact isolated chloroplasts in response to osmotic stress and its effect on photosynthesis and chloroplast volume. Plant physiology. 1985;79(4):996-1002.

70. Valerio C, Costa A, Marri L, Issakidis-Bourguet E, Pupillo P, Trost P, et al. Thioredoxin-regulated beta-amylase (BAM1) triggers diurnal starch degradation in guard cells, and in mesophyll cells under osmotic stress. Journal of experimental botany. 2011;62(2):545-55.

71. Xu C, Li X, Zhang L. The effect of calcium chloride on growth, photosynthesis, and antioxidant responses of Zoysia japonica under drought conditions. PloS one. 2013;8(7):e68214.

72. Minchin PE, Thorpe MR, Farrar JF, Koroleva OA. Source-sink coupling in young barley plants and control of phloem loading. Journal of experimental botany. 2002;53(374):1671-6.

73. Hamilton DA, Davies PJ. Mechanism of export of organic material from the developing fruits of pea. Plant physiology. 1988;86(3):956-9.

74. Kerr PS, Rufty TW, Huber SC. Endogenous Rhythms in Photosynthesis, Sucrose Phosphate Synthase Activity, and Stomatal Resistance in Leaves of Soybean (Glycine max [L.] Merr.). Plant physiology. 1985;77(2):275-80.

75. Martins MC, Hejazi M, Fettke J, Steup M, Feil R, Krause U, et al. Feedback inhibition of starch degradation in Arabidopsis leaves mediated by trehalose 6-phosphate. Plant physiology. 2013;163(3):1142-63.

76. Comparot-Moss S, Kotting O, Stettler M, Edner C, Graf A, Weise SE, et al. A putative phosphatase, LSF1, is required for normal starch turnover in Arabidopsis leaves. Plant physiology. 2010;152(2):685-97.

**Supplementary Figures**

Figure S1. Schematic representation of the metabolic reactions in the model. Included are carbon partitioning between chloroplast and cytosol in source tissues and sucrose export to sink tissues. Short names of the key enzymes, regulating the fluxes are shown near the arrows. Metabolite names are abbreviated in circles. Solid lines show reactions and dashed lines the key allosteric regulations. Reactions occurring only during the day are shown in red, reactions taking place only at night in black, and reactions, which are active during day and night, in green. Carbon is fixed in the Calvin-Benson cycle (CBC) during the day to form triose-phosphates (TP). TP are further directed to starch synthesis in the chloroplast or exported to cytosol for synthesis of sucrose. Starch synthesis is regulated by two irreversible enzymes: stromal fructose bisphosphatase (sFBPase) and ADPglucose phosphatase (AGPase). Sucrose synthesis is regulated by two irreversible enzymes: cytosolic FBPase (cFBPase) and sucrose-phosphate synthase (SPS, combined with sucrose-phosphate phosphatase, SPP). cFBPase is involved in sucrose synthesis only during day, when TP is supplied by the CBC. It is allosterically inhibited by F26P, which is synthesised from F6P by the bisfunctional enzyme F6P kinase/F26P phosphatase (F6PK/F26PP). At night sucrose is synthesised from starch degradation, which starts in chloroplasts to produce maltose (M) and glucose (G) and continues in the cytosol. Only one chloroplastic enzyme, β-amylase (bam), is shown for clarity. Reactions downstream of hexose phosphates in the cytosol (G1P, G6P, F6P) are active both during day and night, with SPS being a key irreversible step leading to generation of sucrose, which is further exported to sink tissues. In sink tissues sucrose is converted to hexose-phosphates (HP), which are finally consumed for growth-related processes. Additional diurnal regulation of the above reactions by SnRK1 and OsmK kinases is shown separately on Fig. 1

Figure S2. Schematic illustration of differences between the diurnal mechanisms regulating carbon fluxes assumed in the current model (left) and P2014 (right). For abbreviations see legend to Fig.1. **Right, P2014**: 1) *SnRK1* kinase is represented by one substance (inhibitor *I*), which inhibits 3 processes: sucrose synthesis, starch degradation and sucrose consumption. *I* is inhibited by sink sugars. 2) Sucrose synthesis is stimulated by sink’s demand D. 3) Starch degradation is activated by a component α, which is activated by clock and light. **Left, current model**: 1) *SnRK1* kinase is described separately in source and sink tissues. In sink tissues *SnRK1* is inhibited by sugars. Additionally *SnRK1* is transcriptionally activated by sugar starvation. Sink’s *SnRK1* inhibits consumption of sugars, while in source tissues *SnRK1* inhibits sucrose synthesis. 2) Both sucrose synthesis and starch degradation are indirectly stimulated by sink demand (*T6P_si_*), through activation of osmo-sensitive kinase *OsmK*. *OsmK* is also activated by the clock and light, via *CaK* kinase. 3) starch degradation is activated by *OsmK*. 4) Sugars activate *T6P_si_* by a double negative feed-forward loop: sugars inhibit *SnRK1*, which inhibits *T6P_si_* synthesis. 5) Sugar consumption is regulated by the clock.

Figure S3. Diurnal profiles of model components under different photoperiods. Dashed, solid and dotted lines correspond to 8L:16D, 12L:12D and 16L:8D diurnal cycles respectively. The panels show diurnal kinetics of *OsmK* (magenta) and *CaK* (blue) (a), β (b), *SnRK1_so_* (green) and *SnRK1_si_* (red) (c) and *X* (d)


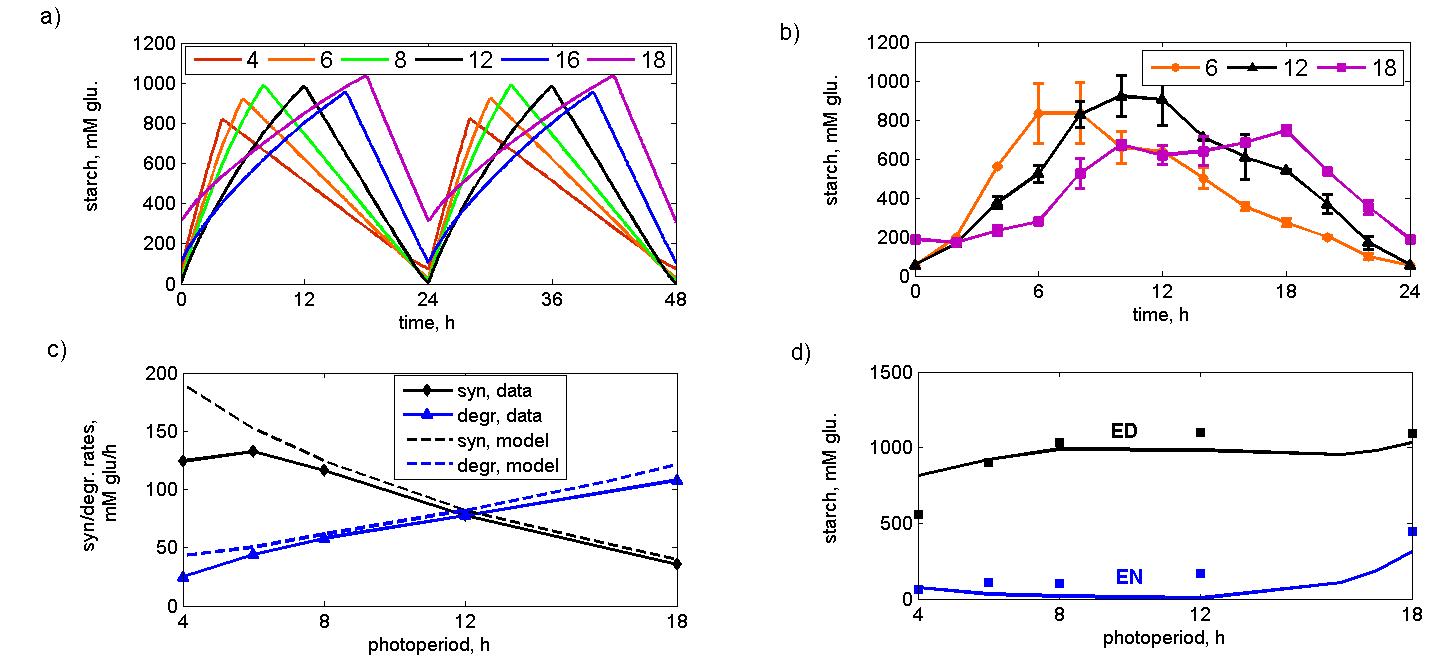


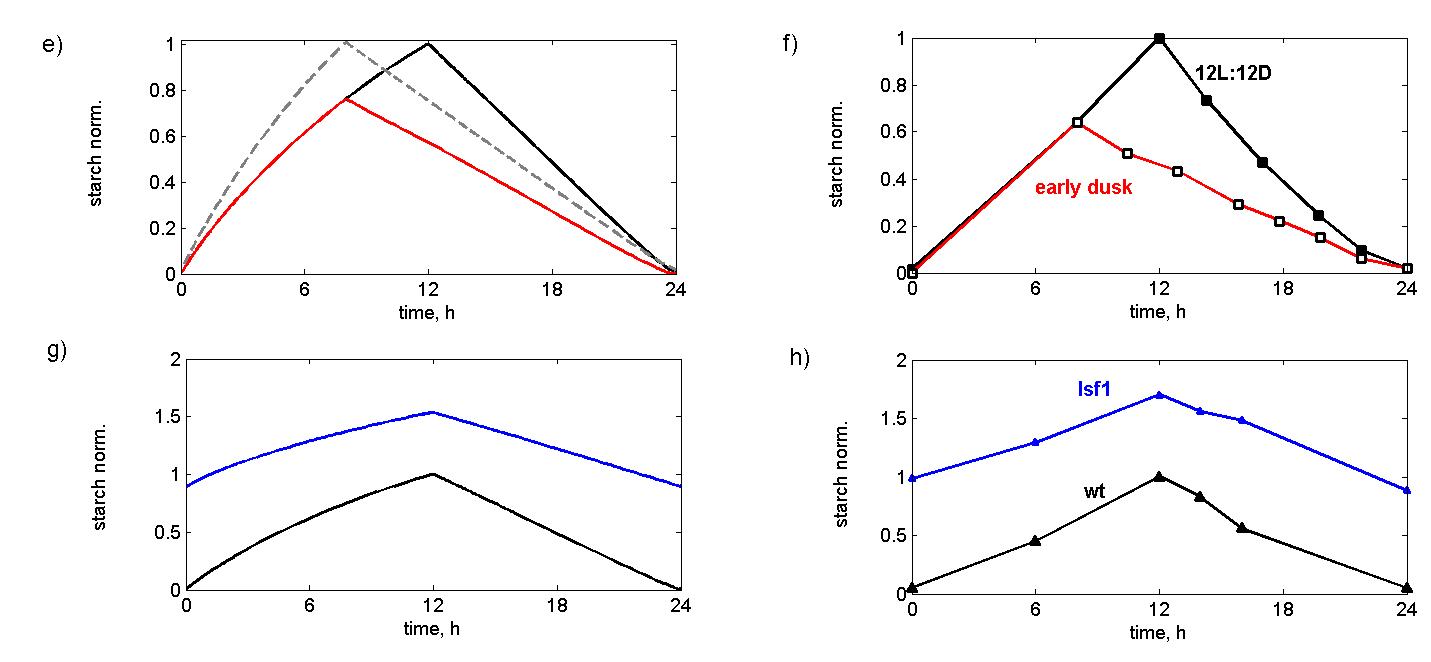


Figure S4. Comparison of the model behaviour with experimental data. a)-d). Starch kinetics under varying photoperiods. a), b). Simulated (a) and experimental (b) timecourses of starch in plants grown under different photoperiods. Duration of photoperiods are shown by numbers in legends: 4 – 4L:20D, 6 – 6L:18D; 8 – 8L:16D; 12 – 12L:12D; 16 – 16L:8D; 18 – 18L:6D. c). Rates of starch synthesis and degradation, shown by black and blue lines respectively. Each data point was derived from an independent experiment with plants grown under different photoperiods. Experimental data and model simulations are shown by solid and dashed lines respectively. d) End of the day (ED) and end of the night (EN) levels of starch under different photoperiods are shown by black and blue colours. Experimental data are shown by symbols and model simulations are show by lines. Data was collected as in c). Experimental plots are redrawn from . e), f) simulated (e) and experimental timecourses of starch in “ealy dusk” experiments, when plants grown under 12L:12D day cycle (black lines) were suddenly exposed to preliminary darkness, 8 h after dawn (red lines). Experimental data are redrawn from . Simulated timecourse of starch in plants grown under 8L:16D day cycle is shown for comparison by grey dashed line on e). Note the changes in starch synthesis rate and the slightly higher level of starch at the end of the night in adopted plants (grey) compared to not-adopted plants (red). g), h). Model simulations (g) and experimental data (h) on starch timecourse in starch-excess mutant *lsf1*, shown by blue lines. Black lines correspond to the wild type. Plants were grown in 12L:12D cycle. Experimental data are redrawn from .

Figure S5. Simulated kinetics of starch under various perturbations of the daily light cycle and circadian clock. a). Simulation of plants grown under so-called “skeleton” photoperiods, with dark pulse being introduced during normal period: 2L:5D:5L:12D. b). Simulation of a “night pulse” experiment, where an additional 5h period of light was inserted 2h after dusk during the normal dark period in 12L:12D cycle. c), d). Simulated changes in starch timecourse in the wild type and mutants of the circadian genes *LHY*, *CCA1*, *PRR9*, *PRR7* and *TOC1*. The wild type, *lhy/cca1*, *prr7/prr9* and *toc1* mutants are shown by black, red, blue and green colors respectively. The *lhy/cca1*, *prr7/prr9* and *toc1* mutants were simulated by putting the rate constants of transcription of respective genes to zero (parameters q_1_, n_1_ for *lhy/cca1*; q_3_, n_4_, n_7_, n_8_, n_9_ for *prr7/prr9*; n_2_ for *toc1*). White and black bars on the x-axis show the light and dark periods respectively. e), f). Simulated kinetics of starch in plants grown under 28h (14L:14D; e) and 20h (10L:10D; f) daily cycles (T cycles). In all plots starch levels are normalized to the peak level in wild type under 12L:12D.

Figure S6. Simulated kinetics of sucrose and starch in P2014. a) Comparison of the model simulations of the total level of sucrose with experimental data for wild type plants. The data points correspond to the data, indicated in the legend of Fig. 2b. Note the too high levels of sucrose in the morning and the too low levels at night in P2014 model in comparison with data. b), c) Simulated timecourses of sucrose and starch in the wild type and *elf3* mutant, shown by black and red lines respectively. Sucrose and starch levels are normalized to their peak levels in wild type. Simulations were performed in 12L:12D diurnal cycle.

Figure S7. Dependence of peak levels of sucrose and *T6P* on the photoperiod. Model simulations of the end-of-the-day (ED) contents of sucrose and *T6P* under different photoperiods are shown by the solid line. Data points are redrawn from (15) and correspond to the ED levels of sucrose and *T6P* under 4L:20D, 6L:18D, 8L:16D, 12L:12D, 18L:6D photoperiods. Concentrations of sucrose and *T6P* under different photoperiods are normalized to the respective 12L:12D values.
