# Supplementary material for: Mathematical modelling of diurnal regulation of carbohydrate allocation by osmo-related processes in plants
Source: J R Soc Interface. 2015 Mar 6;12(104):20141357. doi: 10.1098/rsif.2014.1357 (PMC4345503; doi:10.1098/rsif.2014.1357)
Supplement: Text S2 [file rsif20141357supp2.doc]

**Model files in Matlab**

This file combines the texts of two Matlab files: main program st_P2014.m and ODE equations file Model_st_P2014.m.

**st_P2014.m**

% clock parameters:

m1=0.54; m2=0.24; m3=0.2; m4=0.2; m5=0.3; m6=0.1; m7=0.1; m8=0.5; m9=0.2; m10=0.1; m11=1; m12=1; m13=0.32; m14=0.4; m15=0.7;

m16=0.5; m17=0.5; m18=3.4; m19=0.9; m20=0.6; m21=0.08; m22=0.1; m23=0.1; m24=0.1; m25=0.9; m26=0.5; m27=0.1; m28=28;

m29=0.3; m30=1; m31=0.1; m32=0.2; m33=13; m34=0.6; m35=0.3; m36=0.3; m37=0.4; m38=0.3; m39=0.2;

n1=2.6; n2=0.35; n3=0.29; n4=0.04; n5=0.4; n6=20; n7=0.1; n8=0.5; n9=0.6; n10=0.3; n11=0.6; n12=9; n13=2;

n14=0.1; n15=2; n16=0.1; n17=0.5; n18=0.5; n19=0.2;

p1=0.13; p2=0.27; p3=0.1; p4=0.5; p5=1; p6=0.2; p7=0.3; p8=0.6; p9=0.8; p10=0.54; p11=0.5; p12=10; p13=0.1; p14=0.14; p15=2; p16=0.62;

p17=17; p18=4; p19=1; p20=0.1; p21=1; p22=0.5; p23=0.37; p24=11; p25=2; p26=0.3; p27=0.8; p28=2; p29=0.1; p30=0.9; p31=0.1; p32=0.1; p33=0.2;

g1=0.1; g2=0.01; g3=0.6; g4=0.005; g5=0.2; g6=0.3; g7=1; g8=0.04; g9=0.3; g10=0.5; g11=0.7; g12=0.1; g13=1; g14=0.02; g15=0.4; g16=0.3;

g17=0.6; g18=0.4; g19=0.4; g20=0.03; g21=0.4; g22=0.1; g23=0.4; g24=0.3; g25=0.4; g26=0.3; g27=0.2; g28=0.1; g29=1;

a=2; b=2; c=2; d=2; e=2; ff=2; g=2; h=2; i=2; j=2;

q1=1; q2=1.56; q3=3; Loffset=0; Lamplitude=1; A0=1;

% metabolic parameters:

Vphdat=0.4*3600; Vmfbpases=0.85*3600; Kmfbpase=0.033; KiF6Pfbpase=0.7; Vmagpase=0.24*3600; KmagpG1P=0.08; KmagpATP=0.08; KagpPGA=0.1;

ATPs=0.71; ADPs=0.23; ATPc=2.57; ADPc=0.3; NADP=0.29; NADPH=0.21; Pc=3; PPic=0.04; Ptots=10;

KmATPsynt=0.3; KeqPGI=2.3; KeqPGM=0.058; KeqAld=7.1; KeqIso=22; KeqPGAGAP=0.009;

KtptDHAP=0.077; KtptPi=0.63; KtptPGA=0.25; VmTPT=1.1*3600; KmTHP=0.08; KmHP=0.04;

st0=0.01; f=0.582; fM=0.87; fG3=0.13; KMb=1.46; KMisa=1.46; KMr=12; Kisa0=1.58; Kbg=1.25; KMbg=4.27;

Kdpeeq=1; vc=23; vs=65; Kmex=5.96; KMmex=4; KMglut=19.3; KMhxk=0.035;

KMF6P=2; KMUDPG=2; Vmpgm=1.8*3600; Vmpgi=0.7*3600; KMG6P=6; Vmugpase=3.6*3600; UTPc=1.9; KeqUGPase=0.31; KmUTP=0.093;

Vmdpe1=0.1*3600; Vmdpe2=0.1*3600; Vmhxk=0.1*3600; Vmisa=0.0025*3600; Vmb=0.2*3600; VmPFP=0.6*3600; KmF26PK=0.5; KmF26PP=0.032;

K_F26PK=0.00002*3600; K_F26PP=0.0002*3600; VmGPT=0.0004*3600; Vmresp=0.01*3600; Vmexp=0.004*3600; Vmfbpasec=0.3*3600;

KiF6P_F26P=0.1; Kmfbpasec=0.0025; KmTHPc=0.13; KiG6P=4;

Vmmex=0.2*3600; Vmglut=0.1*3600; KmSP=0.6; KeqPFP=3.3; KiPGA=0.084; KiF26P=0.002; kd_int=0.003*3600;

KeqSuSy=0.15; Vmsps=0.3*3600; Vso=3/4; Vsi=1/4; Ki_consSnRK1=0.01; Ki_consEC=0.04; Ka_consLHY=0.1;

Vmcons=0.3*3600; Vm_St_sink=0.005*3600;

kSnRK1=0.1; Ki_starv=0.03; kIstarv=0.3; Kstarv=4;

ksCaK=1.9; kdCaK=0.4; KdCaK=0.07; KiLHY=0.1;

Vmsuc_hydr_si=200; Vmsuc_syn_si=200; Vmsuc_hydr_so=4;

% parameters of diurnal regulation:

kOsmK=0.5; kaTPS1=2; kaTPS2=5; kdTPS=10; KiTPS=0.01; kaT6P=2; ka_SnRK1si=5;

VmGPT2=0.08*3600; KmGPT2=1; ksGPT=4; kdGPT=0.3; KsGPT=2.4;

Ksb1=0.4; Ksb2=0.2; ksb1=0.02; ksb2=0.06; kdb=0.5; kdX=10; ksX=0.0011;

kidiurn=0.06;

t_T6P=[0 4 8 12 16 20 24];

T6P_w=[0.07 0.09 0.06 0.12 0.18 0.15 0.08]*1000*2/23; %data Wahl 13

t_t6p_w=[0 4 8 12 16 20 24 28 32 36 40 44 48];

t6p_w=[0.07 0.09 0.06 0.12 0.18 0.15 0.08 0.11 0.08 0.12 0.28 0.14 0.06]*1000*2/23;

T6P_Yadav_8=[0.2505 0.29 0.2903 0.2805]/0.52; %data Yadav 13

suc_Yadav_8=[1.163 1.164 1.27 1.32]/1.89;

T6P_Yadav_12=[0.34 0.41 0.38 0.5 0.46]/0.52;

suc_Yadav_12=[1.51 1.545 1.655 2.036 2.164]/1.89;

T6P_Yadav_16=[0.52 0.56 0.53 0.65 0.77]/0.52;

suc_Yadav_16=[1.89 2.072 2.2 2.42 2.49]/1.89;

T6P_mod=[6.09 11.42 17.97]/11.42; % model simulations of Yadav 13, end-of-the-day T6P and suc

suc_mod=[39.55 55.11 62.82]/55.11;

%figure(40)

%plot(T6P_Yadav_8,suc_Yadav_8,'LineStyle','-','Marker','square','MarkerEdgeColor','k','MarkerFaceColor','k','MarkerSize',6);

%hold on;

%plot(T6P_Yadav_12,suc_Yadav_12,'LineStyle','-','Marker','diamond','MarkerEdgeColor','k','MarkerFaceColor','k','MarkerSize',6);

%hold on;

%plot(T6P_Yadav_16,suc_Yadav_16,'LineStyle','-','Marker','square','MarkerEdgeColor','k','MarkerFaceColor','k','MarkerSize',6);

%hold on;

%plot(T6P_mod,suc_mod,'LineStyle','-','Marker','square','MarkerEdgeColor','b','MarkerFaceColor','g','MarkerSize',6);

%hold on;

t_suc_Martins=[0 4 8 11.68 12.38 13 14 15 16 17.87 21 23.77]; % data Martins 13

suc_Martins=[1.324 2.332 2.473 3.385 1.938 1.6 2.37 3.09 2.585 2.463 1.592 1.758]/3.385;

t_suc_ComparotMoss=[0 6 12 14 18 24]; % data Comparot-Moss 10

suc_ComparotMoss=[0.66 1.49 1.57 1.21 1.41 0.76]/1.57;

t_elf3=[0 2 12 22 24]; % data Yazdanbakhsh 11

st_elf3=[3.88 7.54 19.235 7.166 4]/17.35;

st_elf3_er=[0.7 0.8 3.5 1.4 0.7]/17.35;

st_wt=[1.53 1.89 17.35 3.05 1.65]/17.35;

st_wt_er=[0.7 0.6 0.9 0.5 0.6]/17.35;

%figure(40)

%errorbar(t_elf3,st_wt,st_wt_er);

%hold on;

%errorbar(t_elf3,st_elf3,st_elf3_er);

%hold on;

%Vso=3/8; % for defoliation simulations

dawn1=0; % for skeleon photoperiod simulations

dusk1=2;

dawn2=7;

dusk2=12;

period=24;

dawn=0;

dusk=12;

dusks=dusk;

dawns=dawn;

day_numb=1;

% y(1) LHY mRNA

% y(2) P

% y(3) GI-ZTL2

% y(4) GI-ELF3 cytoplasm

% y(5) LHY prot

% y(6) TOC1 mRNA

% y(7) PRR9 prot

% y(8) PRR5 (NI) mRNA

% y(9) PRR5 (NI) prot

% y(10) GI prot cytoplasm

% y(11) TOC1 prot

% y(12) ZTL

% y(13) EC

% y(14) GI mRNA

% y(15) PRR9 mRNA

% y(16) PRR7 mRNA

% y(17) PRR7 prot

% y(18) ELF4 mRNA

% y(19) ELF4 prot

% y(20) LHY prot modif.

% y(21) ABAR mRNA

% y(22) COP1 cytoplasm

% y(23) ELF3 mRNA

% y(24) ELF3 cytoplasm

% y(25) ELF3 nuclear

% y(26) COP1 nuclear night

% y(27) COP1 nuclear day

% y(28) LUX mRNA

% y(29) LUX prot

% y(30) ABAR prot

% y(31) SnRK2 prot

% y(32) X

% y(33) TPSsi

% y(34) TPSso

% y(35) betta

% y(36) GPT2

% y(37) St_isa_int (integral amount of starch linkage groups released by ISA in dark)

% y(38) St_bam_int (integral amount of starch degraded by b amylase)

% y(39) F26P

% y(40)-G2 cytosol

% y(41)-G cytosol

% y(42)-sucrose cytosol

% y(43)-THP stroma

% y(44)-HP stroma

% y(45)-Pi stroma

% y(46)-G1P cytosol

% y(47)-G6P cytosol

% y(48)-F6P cytosol

% y(49)-UDPG cytosol

% y(50)-THP cytosol

% y(51)- starch

% y(52)- GN - starch linkage groups

% y(53)- G2 maltose stroma

% y(54)- G3 stroma

% y(55)- G stroma

% y(56)- G5 stroma

% y(57)- HP sink

% y(58)- sucrose sink

% y(59)- snrk1 source

% y(60)- CaK

% y(61)- starvation sensor

%Solving the ODEs and draw traectories

y0=[0.963996528217875,0.955930331233113,0.0869579361032841,0.0178985412288256,0.591055064698317,0.0413890639046443,0.0248603017870938,0.159693698795584,0.0903363573477447,0.0124484542660736,0.0609868860545552,0.252915742068557,0.126958526699812,0.138998272337330,0.0706986522777157,0.292177728289056,0.179819420763955,0.199626854914636,0.329612824334539,0.0951687042525368,0.941783212019735,1.31427314091740,0.245058667174315,0.140863147698434,0.309240374125784,0.843899350438395,0.407323245974141,0.0761960299239006,0.439495163003609,0.432694454265046,0.203314864964534,0.823242355126649,3.27112458604178,0.935362875197377,0.501075337322423,2.76778052129834e-05,82.0633583962270,731.200089834096,0.0357288637372771,0.0703798330301697,0.0198345750991326,24.9312934659740,0.891915351509505,2.25254209388361,1.94031152267906,0.388397169697439,6.71746319155224,2.97836027787515,5.51938255457023,1.36574440799619,6.25164863354173,0.0237022187975357,0.514564149516265,0.417211067267006,0.756871067145447,0.0938709828241497,10.1081249662655,12.7102316172871,0.234500295636774,2.68412299238195,0.979359149592438];

% y0=[0.850918248397059,0.974804193505731,0.0297881677215735,0.0154499073079052,0.612557674868320,0.0324055256986605,0.0291328607670610,0.223362091377976,0.124664664679367,0.0129475963659330,0.0488549907226732,0.239684092799563,0.105125447914267,0.149829507814430,0.0764885782631699,0.385430321604646,0.257881020246536,0.191890046653075,0.302695321723405,0.113149531115642,1.01409075623449,1.32446813870252,0.211071578014438,0.123467624034890,0.294274835518306,0.894121849600436,0.441212165902626,0.0736758376287503,0.383995812563510,0.372269914632015,0.232945464339383,0.601442593383991,1.92891694054532,0.546653482290842,0.750518649347473,0.000130047746338723,80.3572135925841,729.497019627284,0.0960002757348208,0.0824835048687258,0.0369827534833363,24.6779177453370,0.751709622186829,4.45618860482275,1.16139042377917,0.601057490287386,10.3759819239820,4.54661900655765,8.71086092746686,1.96013601713458,18.1158181372551,0.0228740361233219,0.547329588735173,0.511166784900273,0.849664580258239,0.129513218760080,13.3093308139733,15.3983078607070,0.422785624055075,2.66603639693158,0.990655137117282;]; % 8L:16D

% y0=[1.00835952374165,0.893280641240729,0.205847857773525,0.0262880441381559,0.504353867627644,0.0637984320967223,0.0227879076570213,0.0887764780623917,0.0575000241889460,0.0145649228447693,0.0855157364424443,0.278141831017728,0.156313408820468,0.144044546206489,0.0618119135708388,0.165860791940507,0.0925046679001787,0.254925125876355,0.492160921516756,0.0678876097869242,0.814213925698589,1.28055418943646,0.308822620996093,0.157911999301960,0.245058015755773,0.749821844021678,0.336508339108246,0.0998695630486265,0.649602722534303,0.510337600366419,0.178284564650476,1.08880438124614,6.23552907408503,1.54503925695219,0.334332229293963,1.27017840039172e-05,72.6214562815979,634.220400741173,0.0167546318424967,0.258020111571068,0.370396018775683,37.9997711333850,0.931785301354939,1.69169375070256,2.30778637485998,0.379908473554405,6.57797937858273,2.89964671219956,5.27649016351927,1.18604717044760,106.089221215458,0.0784762643943354,1.75680231893923,1.74994758751494,2.47285747677039,0.645685076813981,9.78713398890803,14.6905600823506,0.138497313723228,2.81666931545288,0.953552868844621;]; % 16:8

% y0=[0.240316494847432,0.955930491673673,0.113273809091702,1.66000422680143e-236,0.229998618433846,0.623845056101801,0.0325107202580871,0.0309907516120250,0.0237050549489305,0.190269278782472,0.567422998741056,0.249402135277964,-4.25950218823618e-20,1.06217049327445,0.0405721326202855,0.00734875802996345,0.00664685683880266,0.697734392111757,0.907429433724229,0.0490624906043135,0.210196914845016,1.31427308725170,5.64686506243485e-236,8.53553509694772e-237,1.18308303201510e-237,0.843894807792093,0.407328002635628,0.697734392111583,1.96200958642593,0.822865409990035,0.121047493505068,0.874708699564317,1.72014971667195,0.950017963137938,0.447574216156281,1.68750423239595e-05,87.2368364471566,607.774501258557,0.0376814021634492,0.149290302125341,0.109040764137369,25.4387590697112,0.867446146650161,2.70956655306255,1.75499379659956,0.467479298582056,8.08052503562925,3.55440018254696,6.65499720622189,1.47336131236202,146.254864748850,0.0606919342558584,1.04487629128997,1.24325141182760,1.69100178673516,0.462103988014575,7.13289931852590,10.3331722623930,0.228999260654897,3.05206599870360,0.760270855534719]; %elf3

if (y0(43)+y0(44)+y0(45))>Ptots

figure (99);

title({'wrong initial conditions'});

hold on;

end

options=odeset();

t=[0 period*day_numb];

[T, Y] = ode23s(@Model_st_P2014,t,y0,options,q1,q2,q3,p1,p2,p3,p4,p5,p6,p7,p8,p9,p10,p11,p12,p13,p14,p15,p16,p17,p18,p19,p20,p21,p22,p23,p24,p25,p26,p27,p28,p29,p30,p31,p32,p33,m1,m2,m3,m4,m5,m6,m7,m8,m9,m10,m11,m12,m13,m14,m15,m16,m17,m18,m19,m20,m21,m22,m23,m24,m25,m26,m27,m28,m29,m30,m31,m32,m33,m34,m35,m36,m37,m38,m39,n1,n2,n3,n4,n5,n6,n7,n8,n9,n10,n11,n12,n13,n14,n15,n16,n17,g1,g2,g3,g4,g5,g6,g7,g8,g9,g10,g11,g12,g13,g14,g15,g16,g17,g18,g19,g20,g21,g22,g23,g24,g25,g26,g27,g28,g29,a,b,c,d,e,ff,g,h,j,dusk,dawn,dawn1,dusk1,dawn2,dusk2,period,Loffset,Lamplitude,A0,st0,f,fM,fG3,KMb,KMisa,KMr,Kbg,KMbg,vc,vs,KMmex,KMglut,KMhxk,Vmb,Vmisa,Vmdpe1,Vmdpe2,Vmmex,Vmglut,Vmhxk,Vphdat,ATPs,ADPs,ATPc,ADPc,NADP,NADPH,Pc,PPic,Ptots,KeqPFP,Vmfbpases,Kmfbpase,KiF6Pfbpase,Vmagpase,KmagpG1P,KmagpATP,KagpPGA,KmATPsynt,KmHP,KmTHP,KmSP,KeqPGI,KeqPGM,KeqAld,KeqIso,KeqPGAGAP,VmTPT,Vmsps,KMF6P,KMUDPG,KiG6P,Vmpgm,Vmpgi,Vmugpase,UTPc,KeqUGPase,KmUTP,Vmresp,KmTHPc,Vmexp,Vmfbpasec,Kmfbpasec,KiF26P,VmPFP,KmF26PK,KmF26PP,K_F26PK,K_F26PP,KiF6P_F26P,KiPGA,Vm_St_sink,Ki_consSnRK1,Ki_consEC,Ka_consLHY,Vmcons,Vmsuc_hydr_si,Vmsuc_hydr_so,kd_int,ksb1,ksb2,Ksb1,Ksb2,kdb,ksX,kdX,ksGPT,kdGPT,KsGPT,kidiurn,ksCaK,kdCaK,KdCaK,kSnRK1,dusks,dawns,VmGPT2,Vso,Vsi,ka_SnRK1si,kaTPS1,kaTPS2,kdTPS,KiTPS,kaT6P,kOsmK,Ki_starv,Vmsuc_syn_si,KiLHY,kIstarv,Kstarv);

L1=[];

tw=0.05;

for i=1:size(T)

Th(i)=T(i);

L1(i)=Loffset+0.5*Lamplitude*((1+tanh((Th(i)-24*floor(Th(i)/24)-dawns)/tw))-(1+tanh((Th(i)-24*floor(Th(i)/24)-dusks)/tw)));

end

SPst=Ptots-Y(:,43)-Y(:,44)-Y(:,45);

F6Pst=Y(:,44)/(1+KeqPGI+KeqPGI*KeqPGM);

G1Pst=Y(:,44)*KeqPGM/(1+KeqPGM+1/KeqPGI);

G6Pst=Y(:,44)/(1+KeqPGM+1/KeqPGI);

A=2*KeqAld/KeqIso;

B=1+1/KeqIso+ADPs*NADP*Y(:,45)/(ATPs*NADPH*KeqIso*KeqPGAGAP);

DHAPst=0.5*(sqrt(4*A*Y(:,43)+B.*B)-B)/A;

GAPst=DHAPst/KeqIso;

FBPst=KeqAld*DHAPst.*DHAPst/KeqIso;

PGAst=DHAPst*ADPs*NADP.*Y(:,45)/(ATPs*NADPH*KeqIso*KeqPGAGAP);

B1=1+1/KeqIso+ADPc*NADP*Pc/(ATPc*NADPH*KeqIso*KeqPGAGAP);

DHAPct=0.5*(sqrt(B1*B1+4*A*Y(:,50))-B1)/A;

FBPct=KeqAld*DHAPct.*DHAPct/KeqIso;

PGAct=DHAPct*ADPc*NADP*Pc/(ATPc*NADPH*KeqIso*KeqPGAGAP);

VTPTDHAP=VmTPT*(DHAPst*Pc-DHAPct.*Y(:,45));

VTPTPGA=VmTPT*(PGAst*Pc-PGAct.*Y(:,45));

VGPT=VmGPT2.*L1'.*Y(:,36).*(Y(:,47).*Y(:,45)-Pc*Y(:,44));

VAGPase=Vmagpase*L1'.*G1Pst*ATPs./((G1Pst+KmagpG1P).*(ATPs+KmagpATP*(1+Y(:,45)./(KagpPGA*PGAst+0.00001))))./(1+Y(:,51).*Y(:,51)/1000000);

VsFBPase=Vmfbpases*FBPst./(FBPst+Kmfbpase*(1+F6Pst/KiF6Pfbpase));

VMEX=Vmmex*(Y(:,53)./(Y(:,53)+KMmex)-Y(:,40)./(Y(:,40)+KMmex));

VGLUT=Vmglut*(Y(:,55)./(Y(:,55)+KMglut)-Y(:,41)./(Y(:,41)+KMglut));

STB=f*Y(:,51);

STISA=(1-f)*Y(:,51);

VB=Vmb*(1-L1');

KBACT=(0.5-atan(10*(Y(:,38)./(f*(Y(:,37)+Y(:,38))+st0)-1))/pi);

VISA=Vmisa*(1-L1').*STISA./(STISA+KMisa).*(0.5-atan(10*(Y(:,37)./((Y(:,37)+Y(:,38))*(1-f)+st0)-1))/pi);

VBAMM=VB.*KBACT*fM.*STB./(fM*STB+KMb*(1+(Y(:,53).*Y(:,53)+Y(:,53).*Y(:,54))/KMr));

VBAMG3=VB.*KBACT*fG3.*STB./(fG3*STB+KMb*(1+(Y(:,53).*Y(:,53)+Y(:,53).*Y(:,54))/KMr));

VST_DEGR=(VISA+VBAMM+VBAMG3).*Y(:,32);

VD=Vmdpe2*(0.5*Pc.*Y(:,40)-Y(:,46).*Y(:,41)/Kdpeeq);

VHXK=Vmhxk*Y(:,41)./(Y(:,41)+KMhxk)./(1+Y(:,47)/KiG6P);

VRES=Vmresp*Y(:,50)./(Y(:,50)+KmTHPc).*(1-L1)';

VEXP=Vmexp*(Y(:,42)-Y(:,58));

Vsuc_hydr_so=Vmsuc_hydr_so*Y(:,42);

ST_SINK=L1'.*(Vm_St_sink+VmGPT2.*Y(:,36)).*Y(:,57)./(1+Y(:,51).*Y(:,51)/1000000);

VST=(VAGPase+ST_SINK);

T6P_SI=kaT6P*Y(:,33);

T6P_SO=kaT6P*Y(:,34);

T6P_tot=(T6P_SI*Vsi+T6P_SO*Vso)/(Vsi+Vso);

suc_tot=Y(:,58)*Vsi+Y(:,42)*Vso;

ISI=ka_SnRK1si*Y(:,59)./(1+Y(:,33)+Y(:,57));

ISO=Y(:,59);

CAK=Y(:,60);

OSMK=CAK+0.5*T6P_SI*Vsi/(Vsi+Vso);

KDIURN1=1*OSMK./(1+ISO/kidiurn);

VSPS=Vmsps*Y(:,48)./(Y(:,48)+KMF6P).*Y(:,49)./(Y(:,49)+KMUDPG).*KDIURN1;

VcFBPase=Vmfbpasec*FBPct./(FBPct+Kmfbpasec*(1+Y(:,48)/KiF6Pfbpase+Y(:,49)/KiF26P));

GN=p28*Y(:,10)./(p29+m19+p17*Y(:,25));

EGN=(p18*Y(:,4)+p17*Y(:,25).*GN)./(m10*Y(:,26)+m9*Y(:,27)+p22);

E34=p25*Y(:,19).*Y(:,25)./(p26*Y(:,29)+p21+m10*Y(:,26)+m9*Y(:,27));

AR=0.5*(A0+Y(:,21)+g29-sqrt((A0+Y(:,21)+g29).^2-4*A0*Y(:,21)));

CONS=Vmcons*Y(:,57)./(1+(ISI.*(1+Y(:,13)/Ki_consEC)./(Ki_consSnRK1*(1+Y(:,5)/Ka_consLHY))).*(ISI.*(1+Y(:,13)/Ki_consEC)./(Ki_consSnRK1*(1+Y(:,5)/Ka_consLHY))));

figure (4)

plot(T,Y(:,52),'k');

hold on;

plot(T,Y(:,53),'b');

hold on;

plot(T,Y(:,54),'m');

hold on;

plot(T,Y(:,55),'g');

hold on;

plot(T,Y(:,56),'r');

hold on;

plot(T,Y(:,40),'b:');

hold on;

plot(T,Y(:,41),'g:');

hold on;

title({'starch degradation: Slg-black; Ms-blue; G3-mag; G-green; G5-red'});

figure (5)

plot(T,Y(:,47),'b:');

hold on;

plot(T,Y(:,48),'b-.');

hold on;

plot(T,Y(:,49),'m');

hold on;

plot(T,Y(:,42),'g');

hold on;

plot(T,Y(:,58),'g:');

hold on;

plot(T,Y(:,50),'k');

hold on;

plot(T,PGAct,'k:');

hold on;

plot(T,Y(:,57),'r');

hold on;

title({'cytosol metabolites: THPc-black; G6Pc-glue dot(F6P-dash)';'UDPG-mag; green-sucrose (dot-sink); HPsink-red'});

figure (6)

plot(T,Y(:,43),'k');

hold on;

plot(T,PGAst,'k:');

hold on;

plot(T,DHAPst,'k-.');

hold on;

plot(T,Y(:,44),'b');

hold on;

plot(T,G6Pst,'b:');

hold on;

plot(T,F6Pst,'b-.');

hold on;

title({'stromal metabolites: THPs-black(PGA-dot;DHAP-dash); HPs-blue(dot-G6P;dash-F6P)'});

figure(7)

plot(T,Y(:,6),'r');

hold on;

plot(T,Y(:,1),'k');

hold on;

plot(T,Y(:,5),'k:');

hold on;

plot(T,Y(:,13),'g');

hold on;

title('clock mRNA: LHY-black (protein-dot), TOC1-red, EC-green');

%figure (8)

%plot(T,suc_tot/55.1,'k');

%hold on;

%plot(t_suc_ComparotMoss,suc_ComparotMoss,'LineStyle','-','Marker','diamond','MarkerEdgeColor','b','MarkerFaceColor','b','MarkerSize',6);

%hold on;

%plot(t_suc_Martins,suc_Martins,'LineStyle','-','Marker','square','MarkerEdgeColor','g','MarkerFaceColor','g','MarkerSize',6);

%hold on;

figure (9)

plot(T,T6P_SI,'b:');

hold on;

plot(T,T6P_SO,'b-.');

hold on;

plot(T,T6P_tot,'b');

hold on;

plot(T,suc_tot,'g');

hold on;

figure(10)

plot(T,Y(:,35),'b');

hold on;

plot(T,Y(:,32),'k:');

hold on;

plot(T,KDIURN1,'c');

hold on;

plot(T,Y(:,61),'k');

hold on;

plot(T,ISO,'r');

hold on;

plot(T,ISI,'r:');

hold on;

title({'diurnal regulation: betta-blue; X-black dot; diurn-cyan; ';'Istarv-black; SnRK1so-red; SnRK1si-red dot;'});

figure(11)

plot(T,Y(:,33),'k:');

hold on;

plot(T,Y(:,34),'k-.');

hold on;

plot(T,CAK,'b');

hold on;

plot(T,OSMK,'m');

hold on;

title({'TPS-black (si-dot, so-dash)';'CaK-blue; OsmK-madenda'});

figure (22)

plot(T,Y(:,51),'k');

hold on;

%plot(T,Y(:,51)/983.57,'k'); %starch normalized

%hold on;

title({'starch'});

**Model_st_P2014.m**

function Func = Model_st_P2014(t,y,q1,q2,q3,p1,p2,p3,p4,p5,p6,p7,p8,p9,p10,p11,p12,p13,p14,p15,p16,p17,p18,p19,p20,p21,p22,p23,p24,p25,p26,p27,p28,p29,p30,p31,p32,p33,m1,m2,m3,m4,m5,m6,m7,m8,m9,m10,m11,m12,m13,m14,m15,m16,m17,m18,m19,m20,m21,m22,m23,m24,m25,m26,m27,m28,m29,m30,m31,m32,m33,m34,m35,m36,m37,m38,m39,n1,n2,n3,n4,n5,n6,n7,n8,n9,n10,n11,n12,n13,n14,n15,n16,n17,g1,g2,g3,g4,g5,g6,g7,g8,g9,g10,g11,g12,g13,g14,g15,g16,g17,g18,g19,g20,g21,g22,g23,g24,g25,g26,g27,g28,g29,a,b,c,d,e,ff,g,h,j,dusk,dawn,dawn1,dusk1,dawn2,dusk2,period,Loffset,Lamplitude,A0,st0,f,fM,fG3,KMb,KMisa,KMr,Kbg,KMbg,vc,vs,KMmex,KMglut,KMhxk,Vmb,Vmisa,Vmdpe1,Vmdpe2,Vmmex,Vmglut,Vmhxk,Vphdat,ATPs,ADPs,ATPc,ADPc,NADP,NADPH,Pc,PPic,Ptots,KeqPFP,Vmfbpases,Kmfbpase,KiF6Pfbpase,Vmagpase,KmagpG1P,KmagpATP,KagpPGA,KmATPsynt,KmHP,KmTHP,KmSP,KeqPGI,KeqPGM,KeqAld,KeqIso,KeqPGAGAP,VmTPT,Vmsps,KMF6P,KMUDPG,KiG6P,Vmpgm,Vmpgi,Vmugpase,UTPc,KeqUGPase,KmUTP,Vmresp,KmTHPc,Vmexp,Vmfbpasec,Kmfbpasec,KiF26P,VmPFP,KmF26PK,KmF26PP,K_F26PK,K_F26PP,KiF6P_F26P,KiPGA,Vm_St_sink,Ki_consSnRK1,Ki_consEC,Ka_consLHY,Vmcons,Vmsuc_hydr_si,Vmsuc_hydr_so,kd_int,ksb1,ksb2,Ksb1,Ksb2,kdb,ksX,kdX,ksGPT,kdGPT,KsGPT,kidiurn,ksCaK,kdCaK,KdCaK,kSnRK1,dusks,dawns,VmGPT2,Vso,Vsi,ka_SnRK1si,kaTPS1,kaTPS2,kdTPS,KiTPS,kaT6P,kOsmK,Ki_starv,Vmsuc_syn_si,KiLHY,kIstarv,Kstarv);

tw=0.05;

L=1;

Lst=1;

L=Loffset+Lamplitude*(0.5*((1+tanh((t-period*floor(t/period)-dawn)/tw))-(1+tanh((t-period*floor(t/period)-dusk)/tw))+(1+tanh((t-period*floor(t/period)-period)/tw))));

%L=0.5*((1+tanh((t-period*floor(t/period)-dawn)/tw))-(1+tanh((t-period*floor(t/period)-dusk)/tw)));

%L=0.5*((1+tanh((t-period*floor(t/period)-dawn1)/0.5))-(1+tanh((t-period*floor(t/period)-dusk1)/0.5))+(1+tanh((t-period*floor(t/period)-dawn2)/0.5))-(1+tanh((t-period*floor(t/period)-dusk2)/0.5))+(1+tanh((t-period*floor(t/period)-period)/0.5))); % skeleton

Lst=L;

%Lst=0.5*((1+tanh((t-period*floor(t/period)-dawns)/tw))-(1+tanh((t-period*floor(t/period)-dusks)/tw))+(1+tanh((t-period*floor(t/period)-period)/tw)));

%Lst=0.5*((1+tanh((t-period*floor(t/period)-dawns)/tw))-(1+tanh((t-period*floor(t/period)-dusks)/tw))); %for simulations with non-photosynthestic light

Func = zeros(61, 1);

% y(1) LHY mRNA

% y(2) P

% y(3) GI-ZTL

% y(4) GI-ELF3 cytoplasm

% y(5) LHY prot

% y(6) TOC1 mRNA

% y(7) PRR9 prot

% y(8) PRR5 (NI) mRNA

% y(9) PRR5 (NI) prot

% y(10) GI prot cytoplasm

% y(11) TOC1 prot

% y(12) ZTL

% y(13) EC

% y(14) GI mRNA

% y(15) PRR9 mRNA

% y(16) PRR7 mRNA

% y(17) PRR7 prot

% y(18) ELF4 mRNA

% y(19) ELF4 prot

% y(20) LHY prot modif.

% y(21) ABAR mRNA

% y(22) COP1 cytoplasm

% y(23) ELF3 mRNA

% y(24) ELF3 cytoplasm

% y(25) ELF3 nuclear

% y(26) COP1 nuclear night

% y(27) COP1 nuclear day

% y(28) LUX mRNA

% y(29) LUX prot

% y(30) ABAR prot

% y(31) SnRK2 prot

% y(32) X

% y(33) TPSsi

% y(34) TPSso

% y(35) betta

% y(36) GPT2

% y(37) St_isa_int (integral amount of starch linkage groups released by ISA in dark)

% y(38) St_bam_int (integral amount of starch degraded by b amylase)

% y(39) F26P

% y(40)-G2 cytosol

% y(41)-G cytosol

% y(42)-sucrose cytosol

% y(43)-THP stroma

% y(44)-HP stroma

% y(45)-Pi stroma

% y(46)-G1P cytosol

% y(47)-G6P cytosol

% y(48)-F6P cytosol

% y(49)-UDPG cytosol

% y(50)-THP cytosol

% y(51)- starch

% y(52)- GN - starch linkage groups

% y(53)- G2 maltose stroma

% y(54)- G3 stroma

% y(55)- G stroma

% y(56)- G5 stroma

% y(57)- HP sink

% y(58)- sucrose sink

% y(59)- snrk1 source

% y(60)- CaK

% y(61)- starvation sensor

Gn=p28*y(10)/(p29+m19+p17*y(25));

EGn=(p18*y(4)+p17*y(25)*Gn)/(m10*y(26)+m9*y(27)+p31);

e34=p25*y(19)*y(25)/(p26*y(29)+p21+m10*y(26)+m9*y(27));

ar=0.5*(A0+y(21)+g29-sqrt((A0+y(21)+g29)^2-4*A0*y(21)));

SPs=Ptots-y(43)-y(44)-y(45);

F6Ps=y(44)/(1+KeqPGI+KeqPGI*KeqPGM);

G1Ps=y(44)*KeqPGM/(1+KeqPGM+1/KeqPGI);

G6Ps=y(44)/(1+KeqPGM+1/KeqPGI);

am=2*KeqAld/KeqIso;

bm=1+1/KeqIso+ADPs*NADP*y(45)/(ATPs*NADPH*KeqIso*KeqPGAGAP);

DHAPs=0.5*(sqrt(bm*bm+4*am*y(43))-bm)/am;

FBPs=KeqAld*DHAPs*DHAPs/KeqIso;

PGAs=DHAPs*ADPs*NADP*y(45)/(ATPs*NADPH*KeqIso*KeqPGAGAP);

Vphsyn=Lst*Vphdat;

Vsfbpase=Vmfbpases*FBPs/(FBPs+Kmfbpase*(1+F6Ps/KiF6Pfbpase));

Vagpase=Vmagpase*Lst*G1Ps*ATPs/((G1Ps+KmagpG1P)*(ATPs+KmagpATP*(1+y(45)/(KagpPGA*PGAs+0.00001))))/(1+(y(51)/1000)^2);

b1=1+1/KeqIso+ADPc*NADP*Pc/(ATPc*NADPH*KeqIso*KeqPGAGAP);

DHAPc=0.5*(sqrt(b1*b1+4*am*y(50))-b1)/am;

FBPc=KeqAld*DHAPc*DHAPc/KeqIso;

PGAc=DHAPc*ADPc*NADP*Pc/(ATPc*NADPH*KeqIso*KeqPGAGAP);

Vcfbpase=Vmfbpasec*FBPc/(FBPc+Kmfbpasec*(1+y(48)/KiF6Pfbpase+y(39)/KiF26P));

Vpfp=VmPFP*(PPic*y(48)-Pc*FBPc/KeqPFP);

VtptDHAP=VmTPT*(DHAPs*Pc-DHAPc*y(45));

VtptPGA=VmTPT*(PGAs*Pc-PGAc*y(45));

Stb=f*y(51);

Stisa=(1-f)*y(51);

Vb=Vmb*(1-Lst)*y(61);

Kbact=(0.5-atan(10*(y(38)/(f*(y(37)+y(38))+st0)-1))/pi);

Visa=Vmisa*(1-Lst)*Stisa/(Stisa+KMisa)*(0.5-atan(10*(y(37)/((y(37)+y(38))*(1-f)+st0)-1))/pi);

VbamM=Vb*Kbact*fM*Stb/(fM*Stb+KMb*(1+(y(53)*y(53)+y(53)*y(54))/KMr));

VbamG3=Vb*Kbact*fG3*Stb/(fG3*Stb+KMb*(1+(y(53)*y(53)+y(53)*y(54))/KMr));

VbamG5=Kbg*Vb*y(56)/(y(56)+KMbg*(1+(y(53)*y(53)+y(53)*y(54))/KMr));

VbamMslg=Kbg*Vb*fM*y(52)/(fM*y(52)+KMbg*(1+(y(53)*y(53)+y(53)*y(54))/KMr));

VbamG3slg=Kbg*Vb*fG3*y(52)/(fG3*y(52)+KMbg*(1+(y(53)*y(53)+y(53)*y(54))/KMr));

Vmex=Vmmex*(y(53)/(y(53)+KMmex)-y(40)/(y(40)+KMmex));

Vglut=Vmglut*(y(55)/(y(55)+KMglut)-y(41)/(y(41)+KMglut));

VdpeG3=Vmdpe1*(y(54)*y(54)/9-0.2*y(56)*y(55));

Vd=Vmdpe2*(0.5*y(40)*Pc-y(46)*y(41));

Vhxk=Vmhxk*y(41)/(y(41)+KMhxk)/(1+y(47)/KiG6P);

Vpgm=Vmpgm*(y(46)/KeqPGM-y(47));

Vpgi=Vmpgi*(y(47)/KeqPGI-y(48));

Vugpase=Vmugpase*(y(46)*UTPc-y(49)*PPic/KeqUGPase)/(UTPc+KmUTP);

Vresp=Vmresp*y(50)/(y(50)+KmTHPc)*(1-L);

Vexp=Vmexp*(y(42)-y(58));

Vst_sink=1*(Vm_St_sink+1*VmGPT2*y(36))*Lst*y(57)/(1+(y(51)/1000)^2);

Vgpt2=VmGPT2*Lst*y(36)*(y(47)*y(45)-Pc*y(44));

T6Psi=kaT6P*y(33);

T6Pso=kaT6P*y(34);

Isi=ka_SnRK1si*y(59)/(1+y(33)+y(57));

Iso=y(59);

Vst_degr=(VbamM+VbamG3+Visa)*y(32);

CaK=y(60);

OsmK=CaK+kOsmK*T6Psi*Vsi/(Vsi+Vso);

Kdiurn1=OsmK/(1+Iso/kidiurn);

Vcons=Vmcons*y(57)/(1+(Isi*(1+y(13)/Ki_consEC)/(Ki_consSnRK1*(1+y(5)/Ka_consLHY)))^2);

Vsps=Vmsps*(y(48)/(y(48)+KMF6P))*(y(49)/(y(49)+KMUDPG))*Kdiurn1;

Vf26pK=K_F26PK*y(48)/(y(48)+KmF26PK*(1+PGAc/KiPGA))-K_F26PP*y(39)/(y(39)+KmF26PP*(1+y(48)/KiF6P_F26P))*Kdiurn1;

Vsuc_hydr_so=Vmsuc_hydr_so*y(42);

Vsuc_hydr_si=Vmsuc_hydr_si*y(58)-Vmsuc_syn_si*y(57);

Func(1) = 1*(q1*L*y(2)+n1)*g1^a/(g1^a+(y(7)+y(17)+y(9)+y(11))^a)-y(1)*(m1*L+m2*(1-L));

Func(2) = p7*(1-L)*(1-y(2))-m11*y(2)*L;

Func(3) = p12*L*y(12)*y(10)-p13*y(3)*(1-L)-m21*y(3);

Func(4) = p17*y(24)*y(10)-m10*y(4)*y(22)-p18*y(4)+p31*EGn;

Func(5) = (p2+p1*L)*(y(1))-m3*y(5)-p3*y(5)^c/(y(5)^c+g3^c);

Func(6) = 1*n2/(1+(y(5)/(g5*(1+(y(31)/g25)^j)))^e)*g4/(g4+y(13))-y(6)*m5;

Func(7) = p8*y(15)-(m13+m22*(1-L))*y(7);

Func(8) = 1*g23^g/(g23^g+y(11)^g)*(n10*y(20)^e/(g12^e+y(20)^e)+n11*y(17)^b/(g13^b+y(17)^b))-m16*y(8);

Func(9) = p10*y(8)-(m17+m24*(1-L))*y(9);

Func(10)= p11*y(14)-m19*y(10)-p12*L*y(12)*y(10)+p13*y(3)*(1-L)-p17*y(24)*y(10)-p28*y(10)+p29*Gn;

Func(11)= p4*(y(6)+n16)-m8*y(11)-(m6+m7*(1-L))*y(11)*(p5*y(12)+y(3));

Func(11)= p4*y(6)-m8*y(11)-(m6+m7*(1-L))*y(11)*(p5*y(12)+y(3));

Func(12)= 1*p14-m20*y(12)-p12*L*y(12)*y(10)+p13*y(3)*(1-L);

Func(13)= p26*y(29)*e34-m10*y(13)*y(26)-m9*y(13)*y(27)-m32*y(13)*(1+p24*L*(EGn+Gn)^d/(g7^d+(EGn+Gn)^d));

Func(14)= 1*g17^g/(g17^g+y(11)^g)*(q2*L*y(2)+g15^e/(g15^e+y(5)^e)*g14/(g14+y(13))*n12)-y(14)*m18;

Func(15)= 1*g18^g/(g18^g+y(11)^g)*(q3*L*y(2)+g8/(g8+y(13))*(n4+n7*y(5)^e/(y(5)^e+g9^e)))-m12*y(15);

Func(16)= 1*g22^g/(g22^g+y(11)^g)*(n8*(y(5)+y(20))^e/(g10^e+(y(5)+y(20))^e)+n9*y(7)^ff/(g11^ff+y(7)^ff))-m14*y(16);

Func(17)= p9*y(16)-y(17)*(m15+m23*(1-L));

Func(18)= 1*n15*g21^g/(g21^g+y(11)^g)*g6^e/(g6^e+y(5)^e)*g20/(g20+y(13))-y(18)*m34;

Func(19)= p23*y(18)-m35*y(19)-p25*y(25)*y(19)+p21*e34;

Func(20)= p3*y(5)^c/(y(5)^c+g3^c)-m4*y(20);

Func(21)= n17*y(5)^e/(y(5)^e+g28^e)*g24^g/(g24^g+y(11)^g)-m37*y(21);

Func(22)= 1*n5-p6*y(22)-m27*y(22)*(1+p15*L);

Func(23)= 1*n3*g16^e/(g16^e+y(5)^e)-m26*y(23);

Func(24)= p16*y(23)-m9*y(24)*y(22)-p17*y(24)*y(10)-p19*y(24)+p20*y(25);

Func(25)= p19*y(24)-p20*y(25)-m10*y(25)*y(26)-m9*y(25)*y(27)-p25*y(25)*y(19)+p21*e34-p17*y(25)*Gn;

Func(26)= p6*y(22)-n6*L*y(2)*y(26)-n14*y(26)-m27*y(26)*(1+p15*L);

Func(27)= 1*(n14*y(26)+n6*L*y(2)*y(26))-m31*(1+m33*(1-L))*y(27);

Func(28)= n13*g19^g/(g19^g+y(11)^g)*g6^e/(g6^e+y(5)^e)*g2/(g2+y(13))-y(28)*m34;

Func(29)= p27*y(28)-m36*y(29)-p26*y(29)*e34;

Func(30)= p33*g27^h/(ar^h+g27^h)-m39*y(30);

Func(31)= p32-m30*y(31)*y(30);

Func(32) = ksX*y(51)*OsmK*Lst-kdX*y(32)*Lst;

Func(33) = kaTPS1+kaTPS2*L-kdTPS*y(33)*(Isi+KiTPS);

Func(34) = kaTPS1+kaTPS2*L-kdTPS*y(34)*(Iso+KiTPS);

Func(35) = (ksb1*Ksb1^2/(y(11)^2+Ksb1^2)+ksb2*y(5)^2/(y(5)^2+Ksb2^2))-kdb*y(35)*Lst;

Func(36) = ksGPT*Lst*Iso^4/(Iso^4+KsGPT^4)-y(36)*kdGPT;

Func(37) = Visa*y(32)-kd_int*y(37)*Lst;

Func(38) = (VbamM+VbamG3)*y(32)-kd_int*y(38)*Lst;

Func(39)= Vf26pK;

Func(40)= Vmex-2*Vd;

Func(41)= Vglut+Vd-Vhxk;

Func(42)= Vsps-Vexp-Vsuc_hydr_so;

Func(43)= Vphsyn*(3*SPs/(SPs+KmSP)-1.5*y(43)/(y(43)+KmTHP))-2*Vsfbpase-VtptDHAP*vc/vs-VtptPGA*vc/vs;

Func(44)= Vsfbpase-Vagpase-0.5*Vphsyn*y(44)/(y(44)+KmHP)+Vgpt2*vc/vs;

Func(45)= VtptDHAP*vc/vs+VtptPGA*vc/vs+Vagpase+Vsfbpase-Vphsyn*y(45)/(y(45)+KmATPsynt)-Vgpt2*vc/vs;

Func(46)= Vd-Vpgm-Vugpase;

Func(47)= Vhxk+Vpgm-Vpgi-Vgpt2;

Func(48)= Vcfbpase-Vpfp+Vpgi-0.5*Vsps+0.5*Vsuc_hydr_so;

Func(49)= Vugpase-0.5*Vsps+0.5*Vsuc_hydr_so;

Func(50)= VtptDHAP+VtptPGA-2*(Vcfbpase-Vpfp)-Vresp;

Func(51) = Vagpase*Vso+Vst_sink*Vsi-Vst_degr;

Func(52) = Visa*y(32)-VbamMslg-VbamG3slg;

Func(53) = VbamM*y(32)+VbamMslg+0.4*VbamG5-Vmex*vc/vs;

Func(54) = VbamG3*y(32)+VbamG3slg+0.6*VbamG5-6*VdpeG3;

Func(55) = VdpeG3-Vglut*vc/vs;

Func(56) = 5*VdpeG3-VbamG5;

Func(57) = Vsuc_hydr_si-Vcons-Vst_sink;

Func(58) = Vexp*Vso/Vsi-Vsuc_hydr_si;

Func(59) = kSnRK1*(y(35)/(1+1/(1+y(61)/Ki_starv))-y(59));

Func(60) = ksCaK*(1+KiLHY^2/(y(5)^2+KiLHY^2))-kdCaK*y(60)*(1+(1-L)/(1+(y(7)/KdCaK)^2));

Func(61) = kIstarv*(L+(1-L)*y(57)^3/(y(57)^3+Kstarv^3)-y(61));
