# Supplementary material for: Mathematical modelling of diurnal regulation of carbohydrate allocation by osmo-related processes in plants
Source: J R Soc Interface. 2015 Mar 6;12(104):20141357. doi: 10.1098/rsif.2014.1357 (PMC4345503; doi:10.1098/rsif.2014.1357)
Supplement: Table S1 [file rsif20141357supp3.doc]

**Table S1**. Values of the model parameters used in ODE equations, described in the Supplementary Text S1. The Table divided on four parts (A-D), which correspond to different blocks of reactions described in Text S1

| 1. Parameters of carbon fixation and starch synthesis reactions | | | | | | | | | |
| --- | --- | --- | --- | --- | --- | --- | --- | --- | --- |
| Parameter | $Pi_{tot}$ | $K_{M\_ATPsynt}$ | | $K_{M\_HP}$ | $K_{M\_THP}$ | | | $K_{M\_SP}$ | $V_{m\_sFBP}$ |
| Value | 10 mM | 0.3 mM | | 0.04 mM | 0.08 mM | | | 0.6 mM | 0.85 mM/s |
| ref |  |  | |  |  | | | varied, text |  |
| Parameter | $K_{iF6P\_FBPase}$ | $K_{M\_sFBP}$ | | $ATP_{s}$ | $ADP_{s}$ | | | $V_{m\_agp}$ | $K_{M\_agp\_G1P}$ |
| Value | 0.7 mM | 0.033 mM | | 0.71 mM | 0.23 mM | | | 0.24 mM/s | 0.08 mM |
| ref |  |  | |  |  | | |  |  |
| Parameter | $K_{M\_agp\_ATP}$ | $K_{a\_agp\_PGA}$ | | $V_{M\_TPT}$ | $K_{eq\_sAld}$ | | | $K_{eq\_TPiso}$ | $K_{eq\_PGA\_GAP}$ |
| Value | 0.08 mM | 0.1 | | 1.1 mM/s | 7.1 mM^-1^ | | | 22 | 0.009 mM |
| ref |  |  | |  |  | | |  |  |
| Parameter | $NADP$ | $NADPH$ | | $K_{eq\_PGI}$ | $K_{eq\_PGM}$ | | | $v_{phsyn}^{obs}$ | $v_{c},v_{s}$ |
| Value | 0.29 mM | 0.21 mM | | 2.3 | 0.058 | | | 0.4 mM/s | 65, 23 µl/gFW |
| ref |  |  | |  |  | | |  |  |
| 1. Parameters of starch degradation reactions | | | | | | | | | |
| Parameter | $f$ | $f_{M}$ | $f_{G3}$ | | $K_{M\beta}^{St}$ | | $k_{\beta}^{G}$ | | $K_{M\beta}^{G}$ |
| Value | 0.582 | 0.87 | 0.13 | | 1.46 mM | | 1.25 | | 4.27 mM |
| ref |  |  |  | |  | |  | |  |
| Parameter | $K_{M\_mex}$ | $K_{M\_glut}$ | $K_{Misa}$ | | $K_{Mr}$ | | $v_{s}$ | | $v_{c}$ |
| Value | 4 mM | 19.3 mM | 1.46 mM | | 12 mM^2^ | | 65 µL/gFW | | 23 µL/gFW |
| ref |  |  | estim., text | |  | |  | |  |
| Parameter | $K_{eq}^{dpe}$ | $K_{eq}^{dpe2\_phs}$ | $V_{M\_dpe1}$ | | $V_{M\_dpe2}$ | | $V_{M\_mex}$ | | $V_{M\_glut}$ |
| Value | 1 | 1 | 0.1 mM/s | | 0.1 mM/s | | 0.2 mM/s | | 0.1 mM/s |
| ref |  |  |  | |  | |  | |  |
| Parameter | $V_{M\_\beta}$ | $V_{M\_isa}$ | $k_{d\_\mathrm{int}}$ | | $K_{M\_hxk}$ | | $K_{iG6P}$ | | $V_{M\_hxk}$ |
| Value | 0.2 mM/s | 0.0025 mM/s | 0.003 s^-1^ mM^-1^ | | 0.035 | | 4 mM | | 0.1 mM/s |
| ref |  |  |  | |  | |  | |  |
| 1. Parameters of sucrose metabolism | | | | | | | | | |
| Parameter | $NADP$ | $NADPH$ | | $ATP_{c}$ | $ADP_{c}$ | | $Pi_{c}$ | | $K_{M\_cFBPase}$ |
| Value | 0.29 mM | 0.21 mM | | 2.57 mM | 0.3 mM | | 3 mM | | 0.0025 mM |
| ref |  |  | |  |  | |  | |  |
| Parameter | $K_{iF6P\_FBPase}$ | $K_{iF26P}$ | | $v_{M\_cFBPase}$ | $v_{M\_PGI}$ | | $v_{M\_PGM}$ | | $K_{eq\_PGI}$ |
| Value | 0.7 mM | 2 µM | | 0.3 mM/s | 0.7 mM/s | | 1.8 mM/s | | 2.3 |
| ref |  |  | |  |  | |  | |  |
| Parameter | $v_{M\_UGPase}$ | $K_{eq\_UGPase}$ | | $K_{M\_UTP}$ | $UTP_{c}$ | | $K_{M\_F6PK}$ | | $v_{M\_PFP}$ |
| Value | 3.6 mM/s | 0.31 | | 0.093 mM | 1.9 mM | | 0.5 mM | | 0.6 mM/s |
| ref |  |  | |  |  | |  | |  |
| Parameter | $K_{eq\_PGM}$ | $v_{M\_SPS}$ | | $K_{M\_F6P}$ | $K_{M\_UDPG}$ | | $K_{iF6P\_F26PP}$ | | $K_{iPGA}$ |
| Value | 0.058 | 0.3 mM/s | | 2 mM | 2 mM | | 0.1 mM | | 0.084 mM |
| ref |  |  | |  |  | |  | |  |
| Parameter | $K_{eq\_sAld}$ | $K_{eq\_TPiso}$ | | $K_{eq\_PGA\_GAP}$ | $K_{eq\_PGI}$ | | $K_{eq\_PGM}$ | | $K_{M\_F26PP}$ |
| Value | 7.1 mM^-1^ | 22 | | 0.009 mM | 2.3 | | 0.058 | | 0.032 mM |
| ref |  |  | |  |  | |  | |  |
| Parameter | $v_{M\_F6PK}$ | $v_{M\_F26PP}$ | | $v_{M\_resp}$ | $K_{M\_resp}$ | | $PPi_{c}$ | | $K_{eq\_PFP}$ |
| Value | 0.00002 s^-1^ | 0.0002 s^-1^ | | 0.01 mM/s | 0.13 mM | | 0.04 mM | | 3.3 |
| ref |  |  | |  | , see text | | estimated, see text | |  |
| 1. Parameters of the model related with sink metabolism and diurnal regulation. The rate constants of the diurnal regulation are slower than the rate constants of the enzymatic reactions, so the diurnal parameters are presented in h^-1^ units for clarity of the Table | | | | | | | | | |
| Parameter | *vM_exp* | *V_si_* | | *V_so_* | | *vM_suc_hydr_so* | *vM_suc_hydr_si* | | *vM_suc_syn_si* |
| Value | 0.004 mM/s | 0.25 | | 0.75 | | 4 h^-1^ | 0.056 s^-1^ | | 0.056 s^-1^ |
| ref |  |  | |  | |  |  | |  |
| Parameter | *k_sSnRK1_* | *Ki_starv* | | *k_sb,1_* | | *k_sb,2_* | *K_sb,1_* | | *K_sb,2_* |
| Value | 0.1 h^-1^ | 0.03 | | 0.02 h^-1^ | | 0.06 h^-1^ | 0.4 | | 0.2 |
| ref | , text | , text | |  | |  |  | |  |
| Parameter | *k_db_* | *k_Istarv_* | | *K_starv_* | | *Ki_diurn* | *k_dCaK_* | | *k_sCaK_* |
| Value | 0.5 h^-1^ | 0.3 h^-1^ | | 4 mM | | 0.06 | 0.4 h^-1^ | | 1.9 h^-1^ |
| ref |  | , text | | , text | | , text | , text | | , text |
| Parameter | *K_iLHY_* | *K_dCaK_* | | *k_OsmK_* | | *Ki_consSnRK1* | *vM_cons* | | *Ki_consEC* |
| Value | 0.1 | 0.07 h^-1^ | | 0.5 µM^-1^ | | 0.01 | 0.3 mM/s | | 0.04 |
| ref | , text |  | | , text | | , text | , text | | , text |
| Parameter | *Ka_consLHY* | *k_aTPS1_* | | *k_aTPS2_* | | *k_dTPS_* | *K_iTPS_* | | *k_dGPT_* |
| Value | 0.1 | 2 h^-1^ | | 5 h^-1^ | | 10 h^-1^ | 0.01 | | 0.3 h^-1^ |
| ref | , text |  | |  | |  |  | |  |
| Parameter | *k_sGPT_* | *VM_GPT2* | | *K_sGPT_* | | *VM_St_sink* | *k_sX_* | | *k_dX_* |
| Value | 4 h^-1^ | 0.08 mM/s | | 2.4 | | 0.005 mM/s | 0.0011 h^-1^ mM^-1^ | | 10 h^-1^ |
| ref |  |  | | , text | |  |  | |  |
| Parameter | *p_0_* | *Ki_St* | | *k_aT6P_* | | *k_aSnRK1si_* | *Ki_SnRK1_HP* | |  |
| Value | 0.00001 | 1 M | | 2 | | 5 | 1 mM | |  |
| ref |  |  | |  | |  |  | |  |

**References**

1. Arnold A, Nikoloski Z. A quantitative comparison of Calvin-Benson cycle models. Trends in plant science. 2011 Dec;16(12):676-83.

2. Aflalo C, Shavit N. Steady state kinetics of photophosphorylation: Limited access of nucleotides to the active site on the ATP synthetase. FEBS Lett. 1983;154:175-9.

3. Woodrow IE, Murphy DJ, Walker DA. Regulation of photosynthetic carbon metabolism. The effect of inorganic phosphate on stromal sedoheptulose-1,7-bisphosphatase. European journal of biochemistry / FEBS. 1983 Apr 15;132(1):121-3.

4. Charles SA, Halliwell B. In: Akoyunoglou G, editor. Photosynthesis. Philadelphia: Balaban; 1981. p. 347-56.

5. Pettersson G, Ryde-Pettersson U. A mathematical model of the Calvin photosynthesis cycle. European journal of biochemistry / FEBS. 1988 Aug 15;175(3):661-72.

6. Charles SA, Halliwell B. Properties of freshly purified and thiol-treated spinach chloroplast fructose bisphosphatase. The Biochemical journal. 1980 Mar 1;185(3):689-93.

7. Arrivault S, Guenther M, Ivakov A, Feil R, Vosloh D, van Dongen JT, et al. Use of reverse-phase liquid chromatography, linked to tandem mass spectrometry, to profile the Calvin cycle and other metabolic intermediates in Arabidopsis rosettes at different carbon dioxide concentrations. Plant J. 2009 Sep;59(5):826-39.

8. Stitt M, Lilley RM, Heldt HW. Adenine nucleotide levels in the cytosol, chloroplasts, and mitochondria of wheat leaf protoplasts. Plant physiology. 1982 Oct;70(4):971-7.

9. Gibon Y, Blaesing OE, Hannemann J, Carillo P, Hohne M, Hendriks JH, et al. A Robot-based platform to measure multiple enzyme activities in Arabidopsis using a set of cycling assays: comparison of changes of enzyme activities and transcript levels during diurnal cycles and in prolonged darkness. Plant Cell. 2004 Dec;16(12):3304-25.

10. Lilley RM, Chon CJ, Mosbach A, Heldt HW. The distribution of metabolites between spinach chloroplasts and medium during photosynthesis in vitro. Biochim Biophys Acta. 1977 May 11;460(2):259-72.

11. Bassham JA, Krause GH. Free energy changes and metabolic regulation in steady-state photosynthetic carbon reduction. Biochim Biophys Acta. 1969 Oct 21;189(2):207-21.

12. Giersch C, Heber U, Kobayashi Y, Inoue Y, Shibata K, Heldt HW. Energy charge, phosphorylation potential and proton motive force in chloroplasts. Biochim Biophys Acta. 1980 Mar 7;590(1):59-73.

13. Pyl ET, Piques M, Ivakov A, Schulze W, Ishihara H, Stitt M, et al. Metabolism and growth in Arabidopsis depend on the daytime temperature but are temperature-compensated against cool nights. Plant Cell. 2012 Jun;24(6):2443-69.

14. Strand A, Zrenner R, Trevanion S, Stitt M, Gustafsson P, Gardestrom P. Decreased expression of two key enzymes in the sucrose biosynthesis pathway, cytosolic fructose-1,6-bisphosphatase and sucrose phosphate synthase, has remarkably different consequences for photosynthetic carbon metabolism in transgenic Arabidopsis thaliana. Plant J. 2000 Sep;23(6):759-70.

15. Winter H, Robinson DG, Heldt HW. Subcellular volumes and metabolite concentrations in spinach leaves. Planta. 1996;193:530-5.

16. Shiraishi F, Kawakami K, Yuasa A, Kojima T, Kusunoki K. Kinetic expression for maltose production from soluble starch by simultaneous use of beta-amylase and debranching enzymes. Biotechnol Bioeng. 1987 Aug 20;30(3):374-80.

17. Nag A, Lunacek M, Graf PA, Chang CH. Kinetic modeling and exploratory numerical simulation of chloroplastic starch degradation. BMC systems biology. 2011;5:94.

18. Weber A, Servaites JC, Geiger DR, Kofler H, Hille D, Groner F, et al. Identification, purification, and molecular cloning of a putative plastidic glucose translocator. Plant Cell. 2000 May;12(5):787-802.

19. Chia T, Thorneycroft D, Chapple A, Messerli G, Chen J, Zeeman SC, et al. A cytosolic glucosyltransferase is required for conversion of starch to sucrose in Arabidopsis leaves at night. Plant J. 2004 Mar;37(6):853-63.

20. Lu Y, Sharkey TD. The role of amylomaltase in maltose metabolism in the cytosol of photosynthetic cells. Planta. 2004 Jan;218(3):466-73.

21. Lu Y, Steichen JM, Weise SE, Sharkey TD. Cellular and organ level localization of maltose in maltose-excess Arabidopsis mutants. Planta. 2006 Sep;224(4):935-43.

22. Heineke D, Kruse A, Flugge UI, Frommer WB, Riesmeier JW, Willmitzer L, et al. Effect of antisense repression of the chloroplast triose-phosphate translocator on photosynthetic metabolism in transgenic potato plants. Planta. 1994;193:174-80.

23. Deuschle K, Chaudhuri B, Okumoto S, Lager I, Lalonde S, Frommer WB. Rapid metabolism of glucose detected with FRET glucose nanosensors in epidermal cells and intact roots of Arabidopsis RNA-silencing mutants. Plant Cell. 2006 Sep;18(9):2314-25.

24. Pokhilko A, Flis A, Sulpice R, Stitt M, Ebenhoh O. Adjustment of carbon fluxes to light conditions regulates the daily turnover of starch in plants: a computational model. Molecular bioSystems. 2014 Mar 4;10(3):613-27.

25. Weise A, Groner F, Sonnewald U, Deppner H, Lerchl J, Hebbeker U, et al. Spinach hexokinase I is located in the outer envelope membrane of plastids. FEBS letters. 1999;461:13-8.

26. Claeyssen E, Rivoal J. Isozymes of plant hexokinase: Occurrence, properties and functions. Phytochemistry. 2007;68:709-31.

27. Foyer C, Spencer C. The relationship between phosphate status and photosynthesis in leaves. Planta. 1986;167:369-75.

28. Zimmermann G, Kelly GJ, Latzko E. Purification and properties of spinach leaf cytoplasmic fructose-1,6-bisphosphatase. The Journal of biological chemistry. 1978 Sep 10;253(17):5952-6.

29. Gardemann A, Heldt AM. Regulatory properties of fructose-1,6-bisphosphate from spinach chloroplasts. Hoppe-Seyler's Zeitschrift fur Physiologische Chemie. 1983;364(9):1127.

30. Stitt M, Mieskes G, Soling HD, Heldt HW. On a possible role of fructose 2,6-bisphosphate in regulating photosynthetic metabolism in leaves. FEBS letters. 1982;145(2):217-22.

31. Huber SC, Bickett DM. Evidence for control of carbon partitioning by fructose 2,6-bisphosphate in spinach leaves. Plant physiology. 1984 Feb;74(2):445-7.

32. Lytovchenko A, Sweetlove L, Pauly M, Fernie AR. The influence of cytosolic phosphoglucomutase on photosynthetic carbohydrate metabolism. Planta. 2002 Oct;215(6):1013-21.

33. Ciereszko I, Johansson H, Kleczkowski LA. Sucrose and light regulation of a cold-inducible UDP-glucose pyrophosphorylase gene via a hexokinase-independent and abscisic acid-insensitive pathway in Arabidopsis. The Biochemical journal. 2001 Feb 15;354(Pt 1):67-72.

34. Lunn JE, ap Rees T. Apparent equilibrium constant and mass-action ratio for sucrose-phosphate synthase in seeds of Pisum sativum. The Biochemical journal. 1990 May 1;267(3):739-43.

35. Elling L. Kinetic characterization of UDP-glucose pyrophosphorylase from germinated barley (malt). Phytochemistry. 1996;42:955-60.

36. Stitt MH, S; Kerr, P. Control of photosynthetic sucrose synthesis. In: Hatch MD, Boardman NK, editors. The Biochemistry of Plants. New York: Academic Press; 1987. p. 327–409.

37. Walker GH, Huber SC. Spinach leaf 6-phosphofructo-2-kinase. FEBS letters. 1987;213:375-80.

38. Colowick SP, Sutherland EW. Polysaccharide synthesis from glucose by means of purified enzymes. J Biol Chem. 1942;144(2):423-35.

39. Stitt M, Wilke I, Feil R, Heldt HW. Coarse control of sucrose-phosphate synthase in leaves. Planta. 1988;174:217-30.

40. Villadsen D, Nielsen TH. N-terminal truncation affects the kinetics and structure of fructose-6-phosphate 2-kinase/fructose-2,6-bisphosphatase from Arabidopsis thaliana. The Biochemical journal. 2001 Nov 1;359(Pt 3):591-7.

41. Markham JE, Kruger NJ. Kinetic properties of bifunctional 6-phosphofructo-2-kinase/fructose-2,6-bisphosphatase from spinach leaves. European journal of biochemistry / FEBS. 2002 Feb;269(4):1267-77.

42. Macdonald FD, Chou Q, Buchanan BB, M. S. Purification and characterization of fructose-2,6-bisphosphate, a substrate-specific cytosolic enzyme from leaves. J Biol Chem. 1989;264(10):5540-4.

43. Dixon LE, Knox K, Kozma-Bognar L, Southern MM, Pokhilko A, Millar AJ. Temporal repression of core circadian genes is mediated through EARLY FLOWERING 3 in Arabidopsis. Curr Biol. 2011 Jan 25;21(2):120-5.

44. Zeeman SC, Rees TA, ;. Changes in carbohydrate metabolism and assimilate export in starch-excess mutants of Arabidopsis. Plant, Cell and Environment. 1999;22:1445-53.

45. Podesta FE, Plaxton WC. Plant cytosolic pyruvate kinase: a kinetic study. Biochim Biophys Acta. 1992 Nov 20;1160(2):213-20.

46. Plaxton WC. The organization and regulation of plant glycolysis. Annu Rev plant Physiol plant Mol Biol. 1996;47:185-214.

47. Nagele T, Henkel S, Hormiller I, Sauter T, Sawodny O, Ederer M, et al. Mathematical modeling of the central carbohydrate metabolism in Arabidopsis reveals a substantial regulatory influence of vacuolar invertase on whole plant carbon metabolism. Plant physiology. 2010 May;153(1):260-72.

48. Nielsen TH, Veierskov B. Regulation of Carbon Partitioning in Source and Sink Leaf Parts in Sweet Pepper (Capsicum annuum L.) Plants : Role of Fructose 2,6-Bisphosphate. Plant physiology. 1990 Jun;93(2):637-41.

49. Sulpice R, Flis A, Ivakov AA, Apelt F, Krohn N, Encke B, et al. Arabidopsis coordinates the diurnal regulation of carbon allocation and growth across a wide range of photoperiods. Molecular plant. 2014 Jan;7(1):137-55.

50. Comparot-Moss S, Kotting O, Stettler M, Edner C, Graf A, Weise SE, et al. A putative phosphatase, LSF1, is required for normal starch turnover in Arabidopsis leaves. Plant physiology. 2010 Feb;152(2):685-97.

51. Gibon Y, Blasing OE, Palacios-Rojas N, Pankovic D, Hendriks JH, Fisahn J, et al. Adjustment of diurnal starch turnover to short days: depletion of sugar during the night leads to a temporary inhibition of carbohydrate utilization, accumulation of sugars and post-translational activation of ADP-glucose pyrophosphorylase in the following light period. Plant J. 2004 Sep;39(6):847-62.

52. Graf A, Schlereth A, Stitt M, Smith AM. Circadian control of carbohydrate availability for growth in Arabidopsis plants at night. Proceedings of the National Academy of Sciences of the United States of America. 2010 May 18;107(20):9458-63.

53. Wahl V, Ponnu J, Schlereth A, Arrivault S, Langenecker T, Franke A, et al. Regulation of flowering by trehalose-6-phosphate signaling in Arabidopsis thaliana. Science. 2013 Feb 8;339(6120):704-7.

54. Martins MC, Hejazi M, Fettke J, Steup M, Feil R, Krause U, et al. Feedback inhibition of starch degradation in Arabidopsis leaves mediated by trehalose 6-phosphate. Plant physiology. 2013 Nov;163(3):1142-63.

55. Kunz HH, Hausler RE, Fettke J, Herbst K, Niewiadomski P, Gierth M, et al. The role of plastidial glucose-6-phosphate/phosphate translocators in vegetative tissues of Arabidopsis thaliana mutants impaired in starch biosynthesis. Plant Biol (Stuttg). 2010 Sep;12 Suppl 1:115-28.

56. Shen W, Reyes MI, Hanley-Bowdoin L. Arabidopsis protein kinases GRIK1 and GRIK2 specifically activate SnRK1 by phosphorylating its activation loop. Plant physiology. 2009 Jun;150(2):996-1005.

57. Zhang Y, Primavesi LF, Jhurreea D, Andralojc PJ, Mitchell RA, Powers SJ, et al. Inhibition of SNF1-related protein kinase1 activity and regulation of metabolic pathways by trehalose-6-phosphate. Plant physiology. 2009 Apr;149(4):1860-71.

58. Nunes C, Primavesi LF, Patel MK, Martinez-Barajas E, Powers SJ, Sagar R, et al. Inhibition of SnRK1 by metabolites: tissue-dependent effects and cooperative inhibition by glucose 1-phosphate in combination with trehalose 6-phosphate. Plant physiology and biochemistry : PPB / Societe francaise de physiologie vegetale. 2013 Feb;63:89-98.
